# CD146^+^ pericyte-like lung cancer brain metastatic stem cells promote tumor angiogenesis through dual regulatory effects on the VEGF/VEGFR axis

**DOI:** 10.7150/thno.122241

**Published:** 2026-01-01

**Authors:** Wenwen Liu, Wenzhe Duan, Shengkai Xia, Ye Liu, Huiying Chu, Kun Liang, Shaobo Fang, Manqing Lin, Song Wei, Xin You, Qiuping Hu, Jingwei Qi, Qi Wang

**Affiliations:** 1 Department of Respiratory Medicine, the Second Affiliated Hospital of Dalian Medical University, 467 Zhongshan Road, Dalian, 116021, China.; 2 Department of Radiotherapy for Oncology, the Second Affiliated Hospital of Dalian Medical University, 467 Zhongshan Road, Dalian, 116021, China.; 3 Department of Respiratory Medicine, Wuhan No. 1 Hospital, Wuhan, Hubei 430022, China.; 4 State Key Laboratory of Molecular Reaction Dynamics, Dalian Institute of Chemical Physics, Chinese Academy of Science, 457 Zhongshan Road, Dalian, 116023, China.; 5 Department of Medical Imaging, the Second Affiliated Hospital of Dalian Medical University, 467 Zhongshan Road, Dalian, 116021, China.; 6 Department of Medical Oncology, Beijing Chest Hospital, Capital Medical University, 9 Beiguan Street, Beijing, 101149, China.; 7 Department of Medicine, Hematology-Oncology Division, David Geffen School of Medicine, University of California, Los Angeles, USA.

**Keywords:** lung cancer brain metastasis, cancer stem cell, tumor microenvironment, angiogenesis, CD146, anti-vascular therapy

## Abstract

**Rationale:** Anti-angiogenic therapy is indispensable for the treatment of non-small cell lung carcinoma (NSCLC)-derived brain metastasis (BrM). Targeting vascular endothelial growth factor (VEGF) and its receptor (VEGFR) is the most effective strategy against angiogenesis. However, bevacizumab (Bev) shows limited therapeutic effects on NSCLC BrM. The plasticity of cancer stem cells (CSCs) has been found to drive therapeutic resistance via the “mimicry” behavior.

**Methods:** CD146^+^ BrM-CSCs were validated in clinical tissues and organoids using immunostaining assays. The ability of CD146^+^ BrM-CSCs to induce angiogenesis was examined using an *ex vivo* multi-organ microfluidic bionic chip and animal models. The effects of CD146 on the VEGF and VEGFR were investigated by RNA-sequencing, molecular dynamics simulation and further cellular and clinical validations. Mechanisms of CD146 upregulation in the brain microenvironment were explored by proteomics, luciferase reporter assay and immunoprecipitation. The anti-vascular efficacy of drugs targeting CD146 on BrM was evaluated in animal studies.

**Results:** BrM-CSCs mimic the pericytes to promote tumor angiogenesis by acquired high expression of CD146 in the brain tumor microenvironment. CD146 exert a dual promotive effect on VEGF/VEGFR2 axis by both up-regulating tumoral VEGF transcription and stabilizing and sensitizing VEGFR2 on endothelial cells as a co-receptor. Secretion of growth arrest specific 6 (GAS6) by the reactive astrocytes led to the CD146 upregulation by activating AXL. Targeting CD146 by imaprelimab or AXL by bemcentinib exhibits more effective anti-angiogenic effects than Bev for BrM *in vivo*. These findings provide novel anti-vascular strategies for BrM.

**Conclusions:** CD146^+^ BrM-CSCs promotes high vascularization of lung cancer brain metastases through dual enhancement of VEGF/VEFGR, which suggests that targeting CD146 is a novel anti-vascular strategy for BrM.

## Introduction

Brain metastasis (BrM) is frequent in lung cancer, and approximately 40-50% of patients with non-small cell lung cancer (NSCLC) develop BrM 1. Patients with BrM face a median 5-year survival rate of less than 5%, largely due to the scarcity of available treatment options 2. Combining anti-angiogenic therapy with immunotherapy or chemotherapy has become an important strategy to treat NSCLC BrM which significantly improves the survival 3. Bevacizumab (Bev), a neutralizing antibody for vascular endothelial growth factor (VEGF), is the first FDA approved and widely used anti-angiogenesis drug. Bev is considered to improve the immunosuppressive microenvironment by inhibiting angiogenesis on the one hand, and to normalize tumor blood vessels to increase drug concentration, thereby enhancing the efficacy of immunotherapy or chemotherapy on the other hand 4. However, the application of Bev is often faced with failure, and revealing its failure causes helps to provide more powerful anti-angiogenesis options for clinical treatment. One possible explanation for the failure of Bev is that the receptor of VEGF (VEGFR) can also be activated by ligands other than VEGF and therefore Bev does not fully inhibit VEGFR activation 5. While many clinical studies have explored the efficacy of inhibitors directly targeting VEGFR 6, the results have not been favorable. Thus, there is an urgent need to seek a strategy that can potently inhibit both VEGF and VEGFR for anti-angiogenic therapy of NSCLC BrM.

Cancer stem cells (CSCs) are a subpopulation of tumor cells with unique abilities for self-renewal and driving tumor diversity and expansion. CSCs often accumulate in perivascular niches and interact closely with the tumor microenvironment (TME). Recently, the plasticity of CSCs, which defines the ability of CSCs to switch between stem-like and differentiated states, has attracted increasing research interest and been found to promote intratumoral heterogeneity and drive tumor relapse, metastasis, and therapeutic resistance. The “mimicry” behavior of CSCs, which suggests that CSCs can impersonate non-tumor cells (such as immune cells, stromal cells, etc.) to support tumor growth, leads to their plasticity 7. A recent study has defined a subpopulation of CD44^+^ lung CSCs with a strong ability to metastasis from in situ to the brain, and this brain metastatic CSC (BrM-CSC) population displays the ability to mimic pericyte cells to promote the colonization of brain metastases 8. However, whether this population of BrM-CSC contributes to the failure of Bev treatment and how BrM-CSCs mimic pericyte cells in the brain microenvironment remains unknown.

In this study, we demonstrated that CD44^+^ BrM-CSCs switch into pericyte-like cells through the acquired overexpression of CD146. CD146^+^ BrM-CSCs exert a comprehensive effect both on the VEGF and VEGFR, and promote tumor angiogenesis. We also investigated the underlying mechanism by which the acquired expression of CD146 in BrM-CSCs is upregulated in the specific cerebral TME and revealed a specific astrocyte-tumor intercellular regulatory axis in which astrocyte-derived growth arrest specific 6 (GAS6) induces the overexpression of CD146 in BrM-CSCs by activating its receptor AXL. Our findings also show that directly targeting CD146+ pericyte-like cells with imaprelimab (an anti-CD146 antibody) or indirectly with bemcentinib (an AXL inhibitor) significantly suppresses intracranial tumor growth and infiltration, and exhibits more effective anti-angiogenic effects than Bev for BrM *in vivo*.

## Materials and Methods

Detailed information on the materials and methods for endothelium conditioned medium collection, endothelial tube formation, adhesion, proliferation, migration and invasion assays, flow cytometry, RNAi and plasmid design and transfection, dual-luciferase reporter assays, and chromatin immunoprecipitation (ChIP) are provided in the Supplemental Data.

### Materials

*Antibodies.* Primary antibodies were used against: CD44 (ab254530, Abcam, RRID:AB_2885131), ABCG2 (27286-1-AP, Proteintech, RRID:AB_2880830), PDGFRβ (3169T, Cell Signaling Technology, RRID:AB_3698544), NG2 (ab275024, Abcam, RRID:AB_2922401), CD31 (ab281583, Abcam, RRID:AB_3096925), CD146 (ab75769, Abcam, RRID:AB_2143375; 17564-1-AP, Proteintech, RRID:AB_2143373), VEGFA (ab214424, Abcam, RRID:AB_3064726; SC-7269, Santa Cruz Biotechnology, RRID:AB_628430), VEGFB (ab133606, Abcam, RRID:AB_3698545), VEGFC (67116-1-IP, Proteintech, RRID:AB_2882420), VEGFR2 (2479, Cell Signaling Technology, RRID:AB_2212507; A16985H, BioLegend, RRID:AB_2728423), phospho-VEGFR2 (3370, Cell Signaling Technology, RRID:AB_2201530), ERK (4695, Cell Signaling Technology, RRID:AB_390779), Phospho-ERK (4370, Cell Signaling Technology, RRID:AB_2315112), MEK (8727, Cell Signaling Technology, RRID:AB_10829473), phospho-MEK (9121, Cell Signaling Technology, RRID:AB_331648), ERK (4695, Cell Signaling Technology, RRID:AB_390779), His-Tag (66005-1-Ig, Proteintech, RRID:AB_11232599), phospho-STAT3 (310019, Zenbio, RRID:AB_3698547), STAT3 (10253-2-AP, Proteintech, RRID:AB_2302876), INSR (ab283689, Abcam, RRID:AB_3676747), HSPG2 (ab255829, Abcam, RRID:AB_3698546), CD80 (66406-1-lg, Proteintech, RRID:AB_2827408), CD206 (18704-1-AP, Proteintech, RRID:AB_10597232), AXL (13196-1-AP, Proteintech, RRID:AB_10642006), phospho-AXL (5724, Cell Signaling Technology, RRID:AB_10544794), GAS6 (13795-1-AP, Proteintech, RRID:AB_2756522), GFAP (23935-1-AP, Proteintech, RRID:AB_2879367), c-Jun (ab40766, Abcam, RRID:AB_731602; ab32137 for ChIP, Abcam, RRID:AB_731608), c-Fos (ab222699, Abcam, RRID:AB_2891049; MA5-15055 for ChIP, Invitrogen, RRID:AB_10984728), GAPDH (140494-1-AP, Proteintech, RRID:AB_2263076), β-actin (20536-1-AP, Proteintech, RRID:AB_10700003). The following secondary antibodies were used: HRP-conjugated Affinipure goat-anti-rabbit IgG second antibody (SA00001-2, Proteintech Group, RRID:AB_2722564), HRP-conjugated IgG Fraction Monoclonal Mouse Anti-Rabbit IgG (SA00001-7L, Proteintech Group, RRID:AB_2890988), CoraLite488-conjugated Goat Anti-Rabbit IgG(H+L) (SA00013-2, Proteintech Group, RRID:AB_2797132). CoraLite® Plus 594-Goat Anti-Rabbit Recombinant Secondary Antibody (RGAR004, Proteintech Group, RRID: AB_3073508), CoraLite® Plus 594-Goat Anti-Mouse Recombinant Secondary Antibody (RGAM004, Proteintech Group, RRID:AB_3073502).

*Reagents.* CellTracker™ CM-Dil (C7000) was bought from Thermo Scientific. DAPI solution (C0060) was bought from Solarbio. Human sCD146 recombination protein (HEK293, his, HY-P756124), Human VEGFA (HY-P78813), Bemcentinib (R428/BGB324, HY-15150 for *in vitro* assays), Stattic (HY-13818) and Bevacizumab (HY-P9906) was bought from MedChemExpress. Ki8751 (S1363) was bought from Selleck. Matrigel (354263) was brought from Corning. Cycloheximide (CHX, A8244) was obtained from ApexBio. Bemcentinib (R428/BGB324, T6269 for animal studies) and Imaprelimab (PRX-003, T77751) was bought from TargetMol. Nuclear extraction kit (P0027) was bought from Beyotime.

### Cell culture

Human non-small cell lung cancer cell lines PC9 (CellCook, Guangzhou, China, cat:CC0204; RRID: CVCL_B260), H2030 (CellCook, Guangzhou, China, cat:CC0242; RRID: CVCL_1517) and H1650 (CellCook, Guangzhou, China, cat:CC0211; RRID: CVCL_1483) were purchased in 2018. Human embryonic kidney cell line 293T (Pricella, Wuhan, China, cat: CL-0005; RRID: CVCL_0063) and human cerebral microvascular endothelial cell line HCMEC/D3 (Pricella, Wuhan, China, cat: CL-0843; RRID: CVCL_U985) were purchased in 2020. Human microglial cell line HMC3 (Pricella, Wuhan, China, cat: CL-0620; RRID: CVCL_II76), human astrocytes HA1800 (Sciencell, USA, cat: #1800) and human microvascular pericyte cells (Pricella, Wuhan, China, cat: CP-H169) were purchased in 2021. Human astrocytes and human microvascular pericyte cells from passages 2 to 5 were used for experiments. All cells tested negative for Mycoplasma contamination and were tested by short tandem repeat (STR) analysis every six months since purchase.

The brain metastatic subpopulations PC9-BrM3, PC9-BrM_chip_ and H2030-BrM were established as previously described 9-11. PC9-CSCs, H1650-CSCs, BrM3-CSCs and BrM_chip_-CSCs labelled by CD44 were isolated by flow cytometric analysis from PC9, H1650, PC9-BrM3 and PC9-BrM_chip_, respectively. The parental PC9 and its derivatives all stably express both green fluorescent protein and luciferase.

RPMI-1640 was used as the culture medium for lung cancer cells. 293T was cultured in DMEM. HMC3 was cultured in MEM. HCMEC/D3, HA1800 and pericytes were respectively cultured in the corresponding media recommended by the manufacturer. The sphere culture of CSCs was performed as previously described 12. All cells were kept in a 37°C humidified environment supplemented with 5% CO2.

### Clinical samples

This study received the approval of the Second Hospital of Dalian Medical University Ethics Review Committee (KY2020-020 for serum, KY2023-140 for cerebrospinal fluid, KY2024-108-01 for tissues and organoids). For organoid generation, primary or brain metastatic tumor tissues were obtained from NSCLC patients with or without BrM by performing surgically resected biopsies. For histological immunostaining, 96 pathologist certified tumor tissues were obtained from lung cancer patients who underwent resection of tumor in situ (n = 52), and lung cancer brain metastasis patients who underwent resection of brain metastases (n = 44) between January 2016 and Octobe 2022. The samples were diagnosed based on pathological assessment. For ELISA assays, serum samples were collected from 160 untreated patients including 113 NSCLC, 23 primary brain tumors (PBT), 24 healthy groups (HG) patients and the 113 NSCLC patients included 22 early lung cancer (ELC), 25 single organ bone metastasis (BoM), 22 single organ live metastasis (LM) and 44 lung cancer BrM (BrM), while cerebrospinal fluid (CSF) samples were collected from 38 untreated BrM patients and 32 untreated patients with disease other than NSCLC BrM. The diagnosis of NSCLC and PBT was confirmed by pathology (surgical resection and/or biopsy). The healthy group consisted of healthy medical examiners who underwent a physical examination. Written informed consent was obtained from all participants.

### Patient-derived tumor organoid cultures

Patient-derived tumor organoids were obtained and cultured as previously described 13. Briefly, fresh tissues were cut into 1 mm^3^ blocks and enzymatically dissociated by dispase (Stemcell, 7923, 37℃, 2 h). The suspension was then passed through a 70 μm filter and centrifuged at 112 rpm for 3 min. The pellet was then resuspended in 50% Matrigel droplets (Corning, 356234), and the solidified Matrigel was preserved in organoid medium (DMEM/F12 with 10% FBS, 50 ng/mL B27, N2, GlutaMAX, EGF, 50 ng/mL bFGF and 10 μM Y-27632, and 1% penicillin-streptomycin, Thermo Scientific), and the medium was renewed every 2-3 days.

### Tumor angiogenesis on a microfluidic chip

A bionic BrM microfluidic chip that allows real-time visual monitoring of the entire BrM process was fabricated as previously described 9. Tumor cells (PC9-CSCs, BrM3-CSCs) were designed to stably express green fluorescent protein, and HCMEC/D3 cells were labeled red with the CellTracker™ CM-Dil dye following the manufacturer's instructions. HCMEC/D3 cells were loaded at a density of 2 × 10^7^ cells/mL into the vascular channels via the inlets while human astrocytes HA-1800 were resuspended in 1:1 (AM) diluted Matrigel and injected into the circular brain parenchyma chamber at a concentration of 10^5^/mL. The loaded cells were incubated in the 37 °C incubator for 4 h. Tumor cells (10^4^ cells in 2 µL) were then injected into the center of the brain parenchyma chamber using a micro-syringe. After 96 h, angiogenesis was observed and images were quantified by AngioTool (RRID:SCR_016393) (https://ccrod.cancer.gov/confluence/display/ROB2/Home) 14.

### Animal studies

Approval for this study was issued by the Dalian Medical University Licensing Committee (AEE23118). Female BALB-c-nu mice (4-6 weeks old, Vital River) were housed in specific pathogen-free conditions.

*Matrigel plugs assays:* Indicated tumor cells (5×10^6^ cells suspended in 100 µL Matrigel 4:1 medium) were injected into mice subcutaneously. For angiogenesis assessment, the subcutaneous tumors were excised after 1 month and IHC was performed. For drug treatments, mice were treated with phosphate-buffered solution (PBS, twice a week, intraperitoneally (i.p.), as the control group^a^), carboxymethylcellulose sodium (CMC-Na, once a day, intragastric administration (i.g.), as the control group^b^), Bev (5 mg/kg, twice a week, i.p.), imaprelimab (10 mg/kg, every two days, i.p.), bemcentinib (75 mg/kg, once a day, intragastric administration (i.g.)), or Bev and imaprelimab/bemcentinib combined, once the tumor reaching a volume of 100 mm^3^. Measurements of tumor volume were taken at three-day intervals, with the volume calculated using the formula: volume (V) = 1/2 × length × width^2^. After 21 days of treatment, animals were examined by a 3.0-T magnetic resonance imaging (MRI) system (Discovery MR750w, GEHealthcare, Chicago, IL, USA) as previously described 15, 16. Animals were subsequently sacrificed and tumors were removed.

*Intracranial tumor model:* After anesthetizing mice with tribromoethanol (10 mL/kg, Sigma), 5×10^5^ indicated tumor cells in 3 µL PBS were implanted into mice by intracranial injection as described previously 17. Brain colonization was analyzed weekly by bioluminescence imaging. Briefly, anaesthetized animals were injected intraperitoneally with D-luciferin (150 mg/kg, Promega) and images were obtained using the IVIS Spectrum Xenogen (PerkinElmer, USA). For angiogenesis assays, at the 6th week after intracranial injection, anaesthetized mice were perfused with PBS and paraformaldehyde; the brain tissues were excised and examined by IHC. For drug treatments, treatments were started at the third week after intracranial injection. Mice were treated with PBS (twice a week, i.p., as the control group), Bev (5 mg/kg, twice a week, i.p.), imaprelimab (10 mg/kg, every two days, i.p.), bemcentinib (75 mg/kg, once a day, intragastric administration (i.g.)), or Bev and imaprelimab/bemcentinib combined. Animals were sacrificed and brain tissues were obtained after 3 weeks of treatment.

### RNA sequencing

RNA sequencing was performed by Novogene Co. Ltd. (Beijing, China). The detailed procedures were presented previously 18.

### Quantitative proteomics

Proteomics analysis was performed by Jingjie PTM BioLab Co. Ltd. (Hangzhou, China). The detailed experimental procedures were presented previously 19.

### Quantitative-polymerase chain reaction (q-PCR)

The detailed experimental procedures were presented previously 19. Primers used in this study are as follows:

*GAPDH*: 5′-CATGAGAAGTATGACAACAGCCT-3′ (forward); 5′-AGTCCTTCCACGATACCAAAGT-3′ (reverse);

*CD146*: 5′-GAAGTCACCGTCCCTGTTTTC-3′ (forward); 5′-CCCCGTTGTCGTTGGTTGT-3′ (reverse).

*VEGFA*: 5′-AGGAGTACCCTGATGAGATCGAGTA-3′ (forward); 5′-TGGTGAGGTTTGATCCGCATA-3′ (reverse).

### Immunohistochemistry (lHC)

The detailed procedures were presented previously 18.

### Confocal immunofluorescence (IF)

The detailed experimental procedures were presented previously 19.

### Western blot analysis

The detailed experimental procedures were presented previously 19.

### Enzyme-linked immunosorbent assay (ELISA)

Sandwich ELISA kits were used to detect sCD146 (OM525744, Omnimabs), VEGFA (OM556320, Omnimabs) and GAS6 (E-EL-H6014, Elabscience) levels in fluid samples following the manufacturer's instructions.

### Co-immunoprecipitation

Cell lysates were incubated with the indicated antibodies and the immunocomplexes were precipitated with protein A/G immunoprecipitation magnetic beads (B23202, Selleck) according to the guidelines. After washing the beads, bound proteins were detected by immune-blotting.

### Preparation of the protein structures

The structures of the VEGFR2 protein (AF-P35968-F1) and sCD146 protein (AF-P43121-F1) were obtained from the AlphaFold Protein Structure Database (https://alphafold.ebi.ac.uk) 20. The D2 to D4 domains of VEGFR2 protein and the D1 to D4 domains were kept. The VEGFR2 structure was aligned to the D2 to D3 domain of VEGFR2 in PDB ID: 3V6B 21, downloaded from the Protein Data Bank (https://www.rcsb.org), to build the extracellular 2:2 VEGFR2/VEGF complex. The structure of VEGFR2 binding with antibody (https://www.rcsb.org, PDB ID: 6LYN) 22 was aligned to the 2:2 VEGFR2/VEGF complex to construct the interaction model between the VEGFR2/VEGF complex and antibody. The structure of sCD146 binding to 2:2 VEGFR2/VEGF complex was obtained by aligning the sCD146 structure to the antibody.

### Molecular dynamics simulations

Atomistic molecular dynamics simulations of the whole extracellular 2:2 VEGFR2/VEGF complex and 2:2 sCD146/VEGFR2/VEGF complex were performed with AMBER16 software, embracing Amber ff14SB force field for protein 23. Each system was put into a TIP3P 24 model hexahedral box, with a distance of 10 Å from the surface of the protein to the edge of the box. Chloride and sodium ions were added to the systems to balance their charges. Energy minimization was conducted for a few thousand steps by imposing a strong restraint on each system. Next, a NVT simulation 25 was performed to heat the whole system slowly from 0 to 300 K, followed by a 5 ns NPT equilibration run. Subsequently, the 1 us production run was performed, and the coordinates of each system were saved every 2 ps (each simulation was repeated five times). During the simulation, all bonds associated with hydrogen atoms were restricted by the SHAKE algorithm. Electrostatic interaction was handled by Particle Mesh Ewald and the cutoff value of nonbonded interactions was set to 9 Å.

### Principal component analysis (PCA)

PCA, a valuable tool for studying conformational changes 26, was used to investigate the extent of correlation motions caused by sCD146 on the VEGFR2/VEGF complex. The PC mode first correlation matrix was computed using the following formula 27:


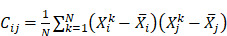

(1)

where *X* is a vector containing the C_α_ atoms Cartesian coordinates of the protein, 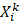
and 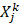
are a pair of vectors *X^k^* to describe the configuration of the system at time step *k*, and 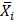, 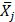
are the average values obtained from the *N* structures extracted from the MD simulation.

### Calculation of binding free energy

The binding free energies between VEGFR2 and VEGF were calculated by the molecular mechanics generalized born surface area (MM/GBSA) method 28 using the 500 snapshots extracted from the last 100ns MD trajectory. The MM/GBSA employed with AMBER16 and the total binding energy (Δ*G_bind_*) 29 was calculated using the following equation:


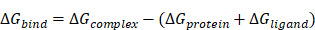

(2)

where Δ*G_bind_* was the binding free energy between VEGFR2 and VEGF, calculated based on the discrepancy between the sum of the free energy of the protein (*G_protein_*) and the total free energy of the complex (*G_complex_*), the protein (*G_protein_*) and the total free energy of the complex (*G_complex_*). The binding energy was computed as:


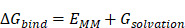

(3)

*E_MM_* represents the molecular mechanical energy of the molecule, which is defined as the sum of the molecule's internal energy, van der Waals energies, and electrostatic energy. Meanwhile, the solvation free energy is described as the sum of the nonpolar and polar contributions to the solvation energy:


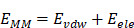

(4)


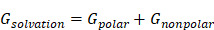

(5)

*G_nonpolar_* was calculated with the LCPO algorithm based on solvent-accessible surface area (SASA) 30:


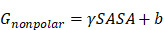

(6)

where *γ* = 0.0072 kcal/mol/Å, and b = 0 kcal/mol 31.

### Statistical analysis

Statistical analysis was performed using GraphPad Prism software (RRID:SCR_002798) 8.0 and IBM Statistical Package for the Social Sciences (SPSS, RRID:SCR_002865) version 25.0 (IBM Corp, Armonk, NY, USA), and the specific statistical tests are indicated in the figure legends. The results of cellular and animal assay show representative samples from three or more replicates, and bar charts show means ± standard deviations (s.d.) from three or more replicates. *p* < 0.05 was considered statistically significant.

## Results

### CD44^+^ lung cancer brain metastatic stem cells mimic pericytes through the acquired overexpression of CD146

**Figure 1:**
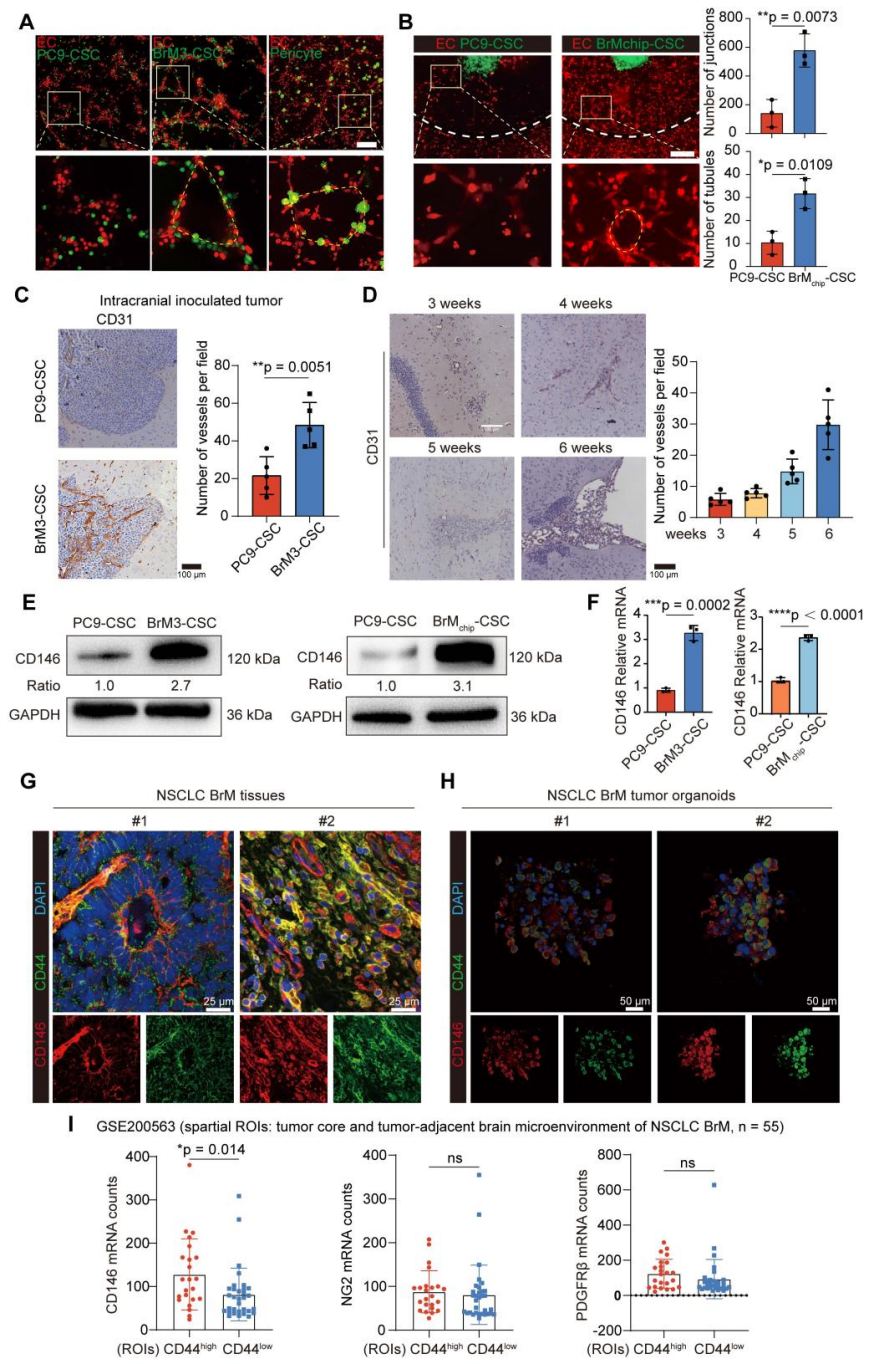
** CD44^+^ lung cancer brain metastatic stem cells mimic pericytes through the acquired overexpression of CD146.** (A) Representative images of PC9-CSCs, BrM3-CSCs or pericytes (green) directly co-cultured with brain microvascular ECs (red) in Matrigel for 12h. Scar bar, 200μm. (B) Angiogenesis of ECs (red) induced by indicated tumor cells (green) on the BrM microfluidic chip. Scar bar, 200μm. Student's *t* test (two-sided). n = 3. (C) PC9-CSCs or BrM3-CSCs were implanted into mice by intracranial injection and the intracranial tumors were excised at the 6th weeks and examined. Images and quantification of vessels in intracranial tumor by CD31 staining. Scar bar, 100μm. Student's *t* test (two-sided). n = 5. (D) Images and quantification of vessels in intracranial tumors which were obtained and stained with CD31 at the 3rd, 4th, 5th and 6th weeks after the mice were intracranially injected with BrM3-CSCs. Scar bar, 100μm. (E) Immunoblots for CD146 in PC9-CSCs, BrM3-CSCs and BrM_chip_-CSCs. (F) qPCR analysis for CD146 in PC9-CSCs, BrM3-CSCs and BrM_chip_-CSCs. Student's *t* test (two-sided). n = 3. (G-H) Immunostaining of CD146 and CD44 in brain metastases tissues (G) and tumor organoids (H) derived from NSCLC BrM patient. (I) Scatter plot analysis for GSE200563 database showing the expression of pericyte markers (CD146, NG2, PDGFRβ) in regions (from tumor core and tumor-adjacent brain microenvironment of NSCLC BrM) with CD44 high expression (CD44^high^, n = 23) and CD44 low expression (CD44^low^, n = 32), respectively. Mann Whitney test (two-sided). ROI, region of interest. Error bars are defined as s.d.

A recent research study demonstrated that pericyte-like cells derived from CD44^+^ lung adenocarcinoma stem cell populations have a strong brain metastasis-initiating capacity 8. We isolated CD44^+^ BrM-CSCs from the previously established brain metastatic subpopulations (BrM) that were derived from a human lung adenocarcinoma cell line PC9 both in a preclinical model (PC9-BrM3, Figure S1A) and a novel bionic microfluidic chip (PC9-BrM_chip_, Figure S1B-C) by flow cytometry 9, 10. These CD44^+^ BrM-CSCs possessed enhanced stemness compared with parental CD44^+^ PC9-CSCs, as evidenced by enhanced stemness biomarker expression, sphere formation ability, and greater self-renewal of metastases *in vivo* (Figure S1D-F). We found that these BrM-CSCs exhibited similar behavioral and physiological functions to pericytes, as evidenced by adhering to ECs and exhibiting strong pro-tubular formation when co-cultured with brain microvascular ECs under three-dimensional (3D) conditions (Figure 1A). Similar results were also observed in the biomimetic microfluidic chip (this organization reflects the 3D formation of BrM including angiogenesis) (Figure 1B). *In vivo* assays also confirmed that BrM-CSCs have a stronger ability to induce angiogenesis compared with PC9-CSCs (Figure 1C and S1G), and the progressive densification of intra-tumoral blood vessels, in parallel with the rapid tumor growth, was observed in BrM-CSC-bearing mice (Figure 1D). Proteomics and further validation showed that CD146, a representative marker of pericytes, showed significant overexpression in BrM-CSCs (Figure 1E-F and S1H-I), while two other biomarkers (NG2, PDGFRβ) of pericytes were negative (Figure S1J). And there was no acquired high expression of CD146 in another lung cancer brain metastatic cell (H2030-BrM), in which CD44 expression was virtually absent (Figure S1K). The existence of the CD44^+^CD146^+^ BrM-CSC subpopulation was further confirmed in lung cancer brain metastatic tissue and patient-derived organoid samples (Figure 1G-H). Analysis of a spatial transcriptomic database of NSCLC BrM 32 also demonstrated that the spatial intra- and peri-tumoral regions of metastases with high CD44 expression had significantly higher expression of CD146 compared with regions with low CD44 expression (Figure 1I). Collectively, those data indicated that CD44^+^ lung cancer BrM-CSCs mimic pericytes through the acquired overexpression of CD146.

### CD146 is overexpressed in NSCLC BrM and correlates with poor prognosis

**Figure 2:**
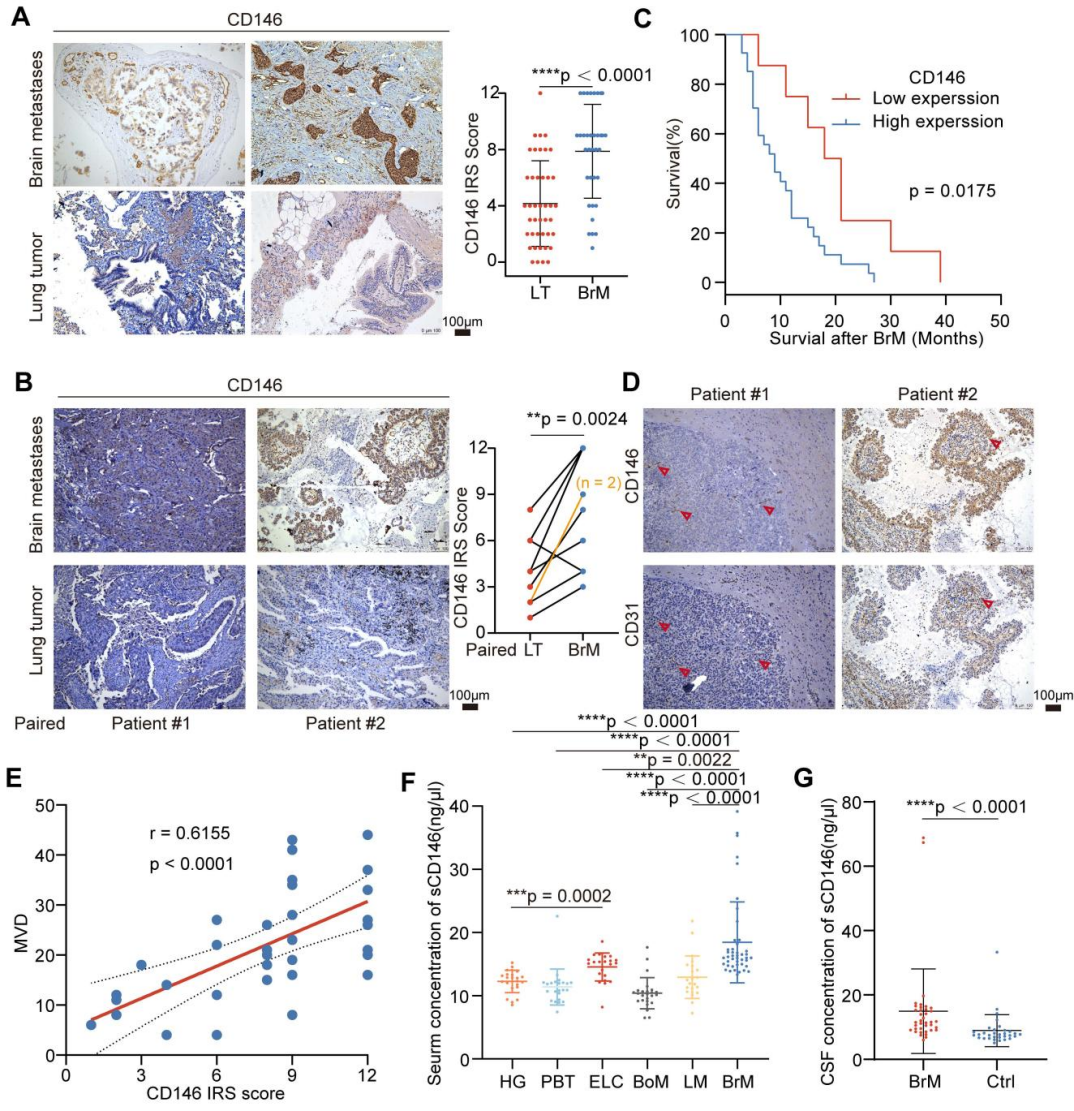
** CD146 is overexpressed in NSCLC BrM and correlates with poor prognosis.** (A) Images and quantification analysis of CD146 staining in primary lung tumor (LT, n = 42) and lung cancer-derived brain metastases (BrM, n = 35) surgical specimens. Student's *t* test (two-sided). (B) 10 of 35 NSCLC patients with BrM underwent orthotopic tumor excision in the early stages of the disease and the primary tumor tissues were obtained. Images and quantification analysis of CD146 staining in brain metastases and the paired tumor lesions *in situ*. Scar bar, 100μm. Student's *t* test (two-sided). (C) The staining results of brain metastases were scored using the immunoreactive score (IRS) established by Remmele and Stegner and categorized into the high expression group (IRS score ≥ 6; n = 27) and the low expression group (IRS score < 6; n = 8). Kaplan-Meier curve analysis of the relationship between CD146 expression and survival after diagnosis of BrM. (D) Representative images of CD146 and CD31 staining in the serial sections of metastasized tumors from lung cancer BrM patients. Scar bar, 100μm. Heterogeneity of CD146 appeared to be consistent with the regional heterogeneity in vascular density distribution indicated by red arrows. (E) A scatter diagram is used to illustrate the correlation between the CD146 staining score and the micro-vascular density (MVD) in the serial sections of metastasized tumors from lung cancer BrM patients. Pearson correlation analysis. n = 35. (F-G) Serum samples were collected from 160 individuals including 113 NSCLC patients (22 early-stage lung cancer, 25 single organ bone metastasis, 22 single organ live metastasis, and 44 lung cancer BrM cases), 23 patients with primary brain tumor, and 24 healthy group individuals. Cerebrospinal fluid (CSF) samples were collected from 38 BrM patients and 32 patients with other diseases as the control group. ELISA results showed the level of sCD146 in the serum samples (F, Mann Whitney test) and CSF samples (G, Mann Whitney test) of participants. HG, healthy group. PBT, primary brain tumor. ELC, early-stage lung cancer. BoM, lung cancer with single organ bone metastasis. LM, lung cancer with single organ bone metastasis. Ctrl, control group. Error bars are defined as s.d.

We next explored the relationship between tumor-derived CD146 expression and prognosis in clinical cohorts of NSCLC BrM. The results of IHC staining showed CD146 expression was higher in the BrM tissues than in the neoplastic tissue *in situ* (Figure 2A-B, Table S1). Survival analysis showed that patients with high CD146 expression had a shorter overall survival (Figure 2C). In addition, since the CD146^+^ BrM-CSCs showed induction and support of neovascularization, we also confirmed a significant correlation between CD146 expression and microvascular density in BrM tissues (Figure 2D-E). Notably, regional heterogeneity in CD146 expression was observed in the same tissue, and this heterogeneity appeared to be consistent with the regional heterogeneity in vascular density distribution (Figure 2D), indicating the close association between tumoral CD146 expression and high vascularization of BrM. Soluble CD146 (sCD146), a form of CD146 generated by ectodomain shedding of membrane CD146, can be detected within the serum derived from cancer patients and the cerebrospinal fluid (CSF) derived from patients with central nervous system diseases and was recently established as a diagnostic and predictive biomarker 33, 34. We found that the levels of sCD146 in the serum and CSF samples were significantly elevated in the BrM group (Figure 2F-G, Table S2-4), indicating the potential role of sCD146 as a liquid biopsy diagnostic marker for NSCLC BrM. Collectively, these data demonstrated that CD146 is overexpressed in NSCLC BrM and correlates with poor prognosis.

### BrM-CSCs promote angiogenesis of BrM via CD146

**Figure 3:**
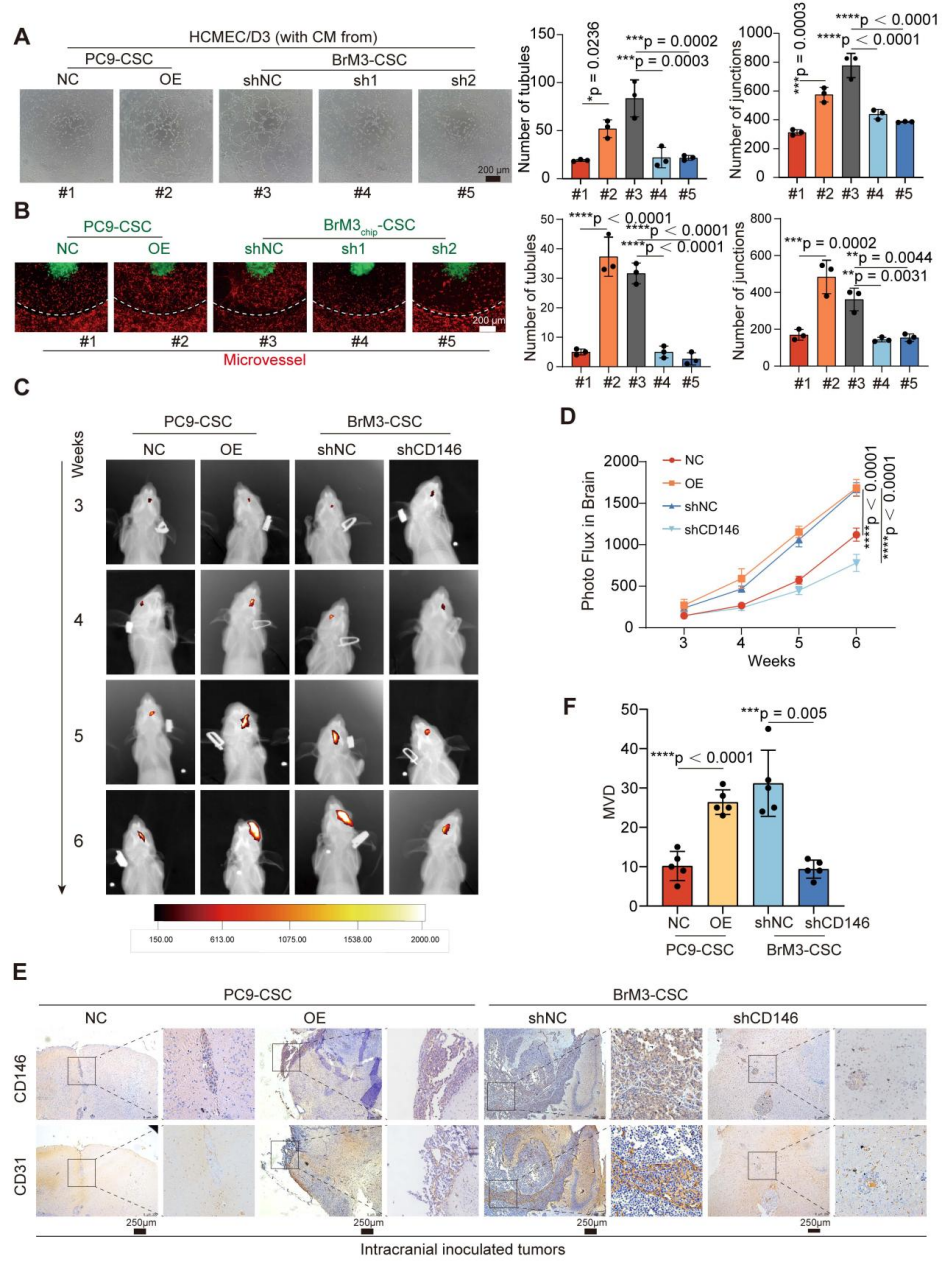
** BrM-CSCs promote angiogenesis of BrM via CD146.** (A) *In vitro* tube formation assays of HCMEC/D3 cells with the treatment of conditioned culture medium (CM) derived from indicated tumor cells. Scar bar, 200μm. One-way ANOVA test. n = 3. (B) Angiogenesis of EC (red) is induced by indicated tumor cells (green) on the BrM microfluidic chip. Scar bar, 200μm. One-way ANOVA test. n = 3. (C) Heat maps are used to visually represent the bioluminescence intensity in representative mice that received intracranial inoculation of the indicated tumor cells. (D) Quantification analysis of bioluminescence readings. Two-way ANOVA test. n = 5. (E-F) Intracranial tumors were obtained and stained with CD146 and CD31, and the micro-vascular densities (MVDs) were quantified and analyzed. Scar bar, 100 μm. Student's *t* test (two-sided). n = 5. NC, negative control plasmid. OE, CD146 plasmid. shNC, negative control shRNA. sh1 and sh2, CD146-targeted shRNA vector 1 and 2. Error bars are defined as s.d.

We silenced the expression of CD146 in BrM3-CSCs and overexpressed CD146 in the parental PC9-CSCs and another lung cancer cell line derived CSCs (H1650-CSCs) (Figure S2A). It was shown that CD146 influenced the vascular adhesion behavior of BrM3-CSCs (Figure S2B). We further assessed the abilities of those CSCs with or without CD146 expression to induce EC angiogenesis. We treated HCMEC/D3 cells with conditioned medium derived from the CSCs *in vitro* and found that tumoral CD146 significantly influenced the tube formation (Figure 3A and S2C), proliferation, migration, and invasion abilities of cells (Figure S2D-F). These behaviors jointly reflect the activities of neovascularization 35. The *ex vivo* bionic BrM microfluidic chip model was also used to assess the ability of tumor cells to induce neovascularization under 3D growth in the brain microenvironment. CSCs with CD146 high expression exhibited a stronger ability to induce new blood vessels derived from the existing microvessel (Figure 3B). Furthermore, *in vivo* animal experiments were performed. Traditional Matrigel plug assays showed that silencing CD146 in BrM3-CSCs cells significantly reduced the vascular density of tumors while overexpressing CD146 in PC9-CSCs cells promoted angiogenesis (Figure S2G). Similar changes in the growth rate and size of subcutaneous tumors were also observed (Figure S2H-I). We also observed the growth and vascularization of BrM tumors *in situ* by intracranial inoculation of tumor cells in nude mice. Consistent with the Matrigel plug assay results, CSCs with high CD146 expression exhibited faster infiltration rates and denser microvascular supplies (Figure 3C-F). Notably, intracranially grown tumors with the same cell inoculation showed more active vascularization than subcutaneous tumors, suggesting that the intracranial microenvironment is supportive of angiogenesis. Taken together, these findings suggested that BrM-CSCs promote angiogenesis of BrM via CD146.

### CD146 exerts a dual promotive effect on the pro-angiogenic VEGFA-VEGFR2 axis

**Figure 4:**
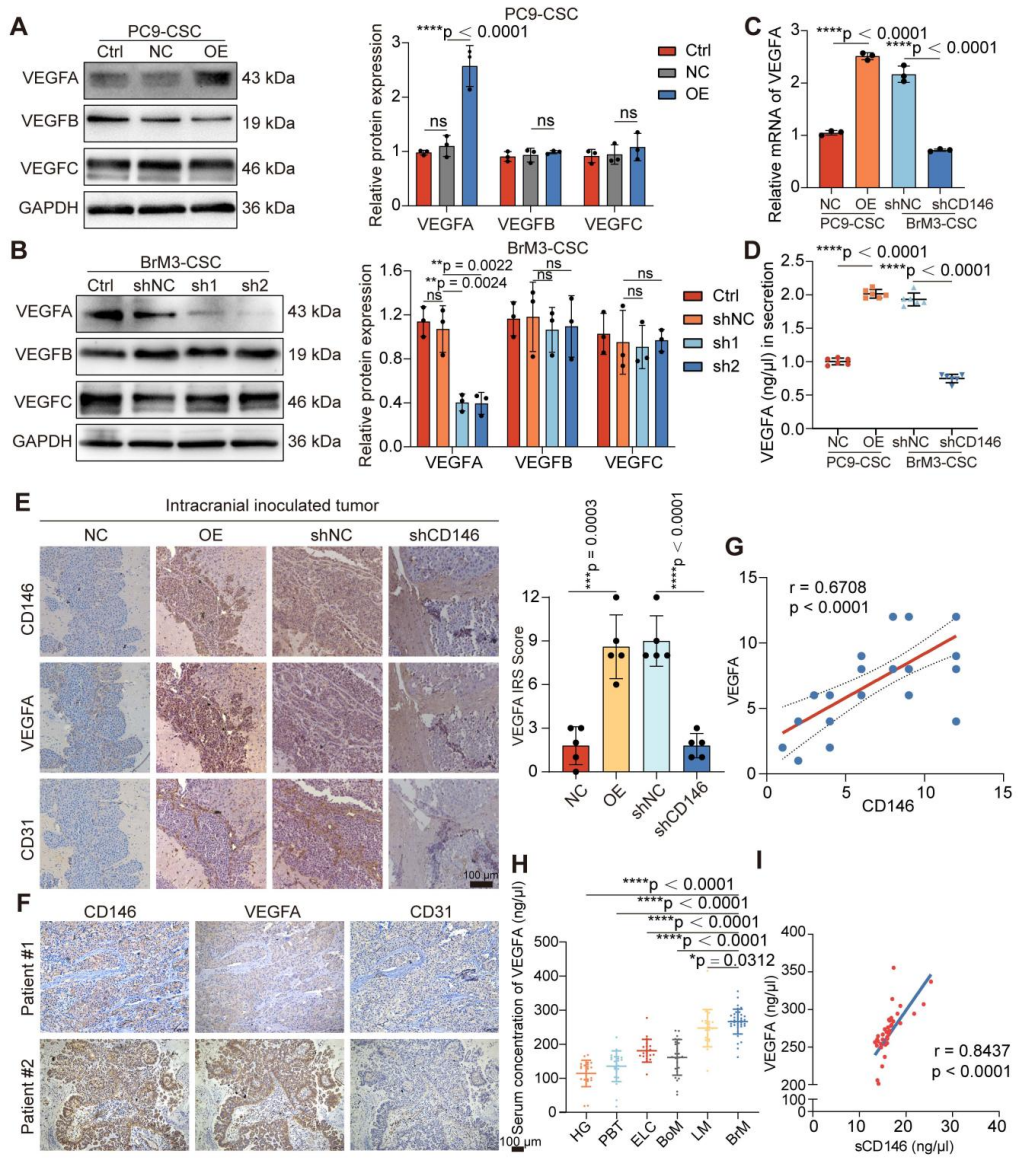
** CD146 upregulates VEGFA in NSCLC BrM-CSCs.** (A-B) The results of western blotting assays showed the expression of VEGFA, VEGFB and VEGFC in indicated cells. Two-way ANOVA test. n = 3. (C) qPCR showed the VEGFA mRNA levels in indicated cells. Student's *t* test (two-sided). n = 3. (D) The results of ELISA showed the concentration of VEGFA in the cell supernatant from indicated cells for 24h culture. Student's *t* test (two-sided). n = 6. (E) Representative images of CD146, VEGFA and CD31 staining in the serial sections of intracranial tumor of mice inoculated with indicated cells, and the IRS scores of VEGFA staining were quantified and analyzed. Scar bar, 100μm. Student's* t* test (two-sided). n = 5. (F) Representative images of CD146, VEGFA and CD31 staining in the serial sections of metastasized tumors from lung cancer BrM patients. (G) Scatter diagram showing the correlation between the CD146 IRS staining scores and the VEGFA IRS staining scores in the serial sections of metastasized tumors from lung cancer BrM patients. Pearson correlation analysis. n = 35. (H) ELISA results showed the level of VEGFA in the serum samples of participants. HG, healthy group (n = 23). PBT, primary brain tumor (n = 23). ELC, early-stage lung cancer (n = 20). BoM, lung cancer with single organ bone metastasis (n = 25). LM, lung cancer with single organ liver metastasis (n = 22). BrM, lung cancer brain metastasis (n = 39). Mann Whitney test. (I) A scatter diagram is used to illustrate the correlation between serum sCD146 and VEGFA levels in lung cancer BrM patients. Two-sided Spearman's correlation analysis. n = 39. NC, negative control plasmid. OE, CD146 plasmid. shNC, negative control shRNA. sh1 and sh2, CD146-targeted shRNA vector 1 and 2. shCD146, CD146-targeted shRNA vector. Error bars are defined as s.d.

RNA-seq in BrM3-CSCs with or without CD146 silencing (BrM3-CSCs-shNC/shCD146) indicated that CD146 plays a major role in the blood vessel morphogenesis pathway and the *VEGFA* gene was identified as one of the differentially expressed genes in this enrichment pathway (Figure S3A). Western blotting (Figure 4A-B), qPCR (Figure 4C), ELISA (Figure 4D), and IHC staining of subcutaneous and intracranial xenograft tumors (Figure 4E and S3B) further confirmed that CD146 significantly enhanced the expression and secretion of VEGFA. We also demonstrated a significant correlation between tumoral CD146 and VEGFA expression in clinical tissue and fluid samples from NSCLC BrM patients (Figure 4F-I).

VEGFA plays a pro-angiogenic role by binding to and phosphorylating VEGFR2 on the surface of ECs, leading to activation of the mitogen-activated protein kinase (MAPK)/extracellular signal-regulated kinase (ERK) signaling pathway 36. We used antibody targeting VEGFA (Bev) and a VEGFR2 inhibitor (ki8751) and observed that the activation of MAPK/ERK signaling and the triggered angiogenesis in ECs induced by PC9-CSCs with CD146 overexpression were abrogated, suggesting that CD146 mediates angiogenesis through VEGFA/VEGFR2 (Figure S4A, C-D). We also supplemented VEGFA in a co-culture system of BrM-CSCs silenced for CD146 with ECs; the inhibited MAPK/ERK activation and the neovascularization in ECs were restored, but only to a partial extent (Figure S4B-D), indicating that CD146 activates ECs not only through VEGFA.

**Figure 5:**
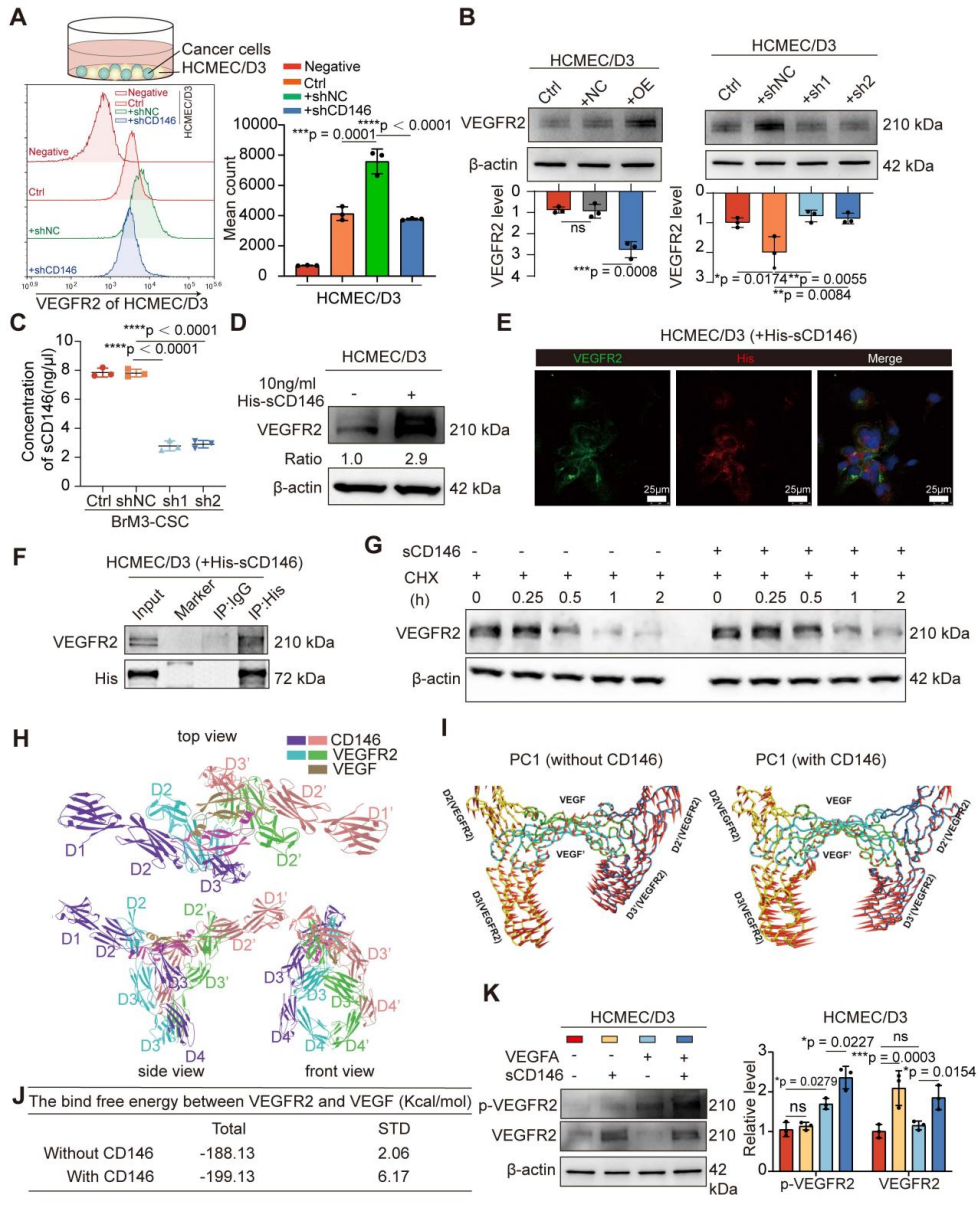
** sCD146 stabilizes and sensitizes VEGFR2 on the surface of cerebral microvascular endothelial cell.** (A) Flow cytometry results showed the membrane VEGFR2 level of HCMEC/D3 cells co-cultured with indicated tumor cells directly. One-way ANOVA test. n = 3. (B) Western blotting assays showed the VEGFR2 level of HCMEC/D3 cells treated with the secretions from indicated tumor cells for 24h. One-way ANOVA test. n = 3. (C) ELISA shows the concentrations of sCD146 in the secretions from indicated tumor cells. One-way ANOVA test. n = 3. (D) Western blotting assays showed the VEGFR2 expression in HCMEC/D3 cell treated with human sCD146 recombinant protein (10 ng/ml for 2h). n = 3. (E) An anti-His antibody was employed in immunoprecipitation to identify the interaction between sCD146 and VEGFR2 in HCMEC/D3 treated with human sCD146 (His) recombinant protein (10 ng/ml for 2h). n = 3. (F) Immunofluorescence was used to analyze the VEGFR2 (green) and His (red) in HCMEC/D3 cells treated with human sCD146 (His) recombinant protein (10 ng/ml for 2h). Scar bar, 25μm. n = 3. (G) Western blotting was used to detect the VEGFR2 levels in individual cell groups treated with CHX (50 μM). n = 3. (H) Interaction mode of sCD146 and VEGFR2/VEGF was shown from the top view, the front view and side view. The two monomers of sCD146 were colored by purple and pink, and the two monomers of VEGFR2 were colored with blue and green. The two monomers of VEGF were colored by olive. (I-J) Comparison of the first principal components of VEGF/VEGFR2 complex in the presence or absence of sCD146. The VEGF/VEGFR2 complex was showed by tube and the arrow represented the trend of protein movement. (K) Western blotting assays showed the levels of VEGFR and p-VEGFR in HCMEC/D3 cell treated with VEGFA (50 ng/ml for 5min), or/and sCD146 (10 ng/ml for 2h). Two-way ANOVA test. n = 3. Ctrl, control group. NC, negative control plasmid. OE, CD146 plasmid. shNC, negative control shRNA. shCD146, CD146-targeted shRNA vector. Error bars are defined as s.d.

We found the presence of BrM-CSCs not only led to high levels of phosphorylated endothelial VEGFR2 but also caused a significant increase in total VEGFR2 expression (Figure S4E). To clarify whether these results were caused by the increased CD146, we directly co-cultured BrM-CSCs (FITC-positive) with or without CD146 silencing with ECs (FITC-negative) and analyzed the expression of VEGFR2 on the membrane surface of the ECs by flow cytometry. The results showed that CD146 silencing attenuated the increase of VEGFR2 on ECs induced by BrM-CSCs (Figure 5A). By administrating the secretions from tumor cells with modulated CD146 gene expression to ECs, we found that secretions from tumor cells with CD146 high expression significantly promoted endothelial VEGFR2 expression whereas secretions from cells with knockdown of CD146 had little effect on VEGFR2 (Figure 5B), and ELISA showed that the level of secreted sCD146 was markedly reduced with CD146 knockdown (Figure 5C). The regulation of VEGFR2 expression in ECs by sCD146 was further demonstrated by the observation that human sCD146 recombinant protein induced endogenous VEGFR2 (Figure 5D).

A previous study showed that CD146 binds to VEGFR2 as a co-receptor on the vascular membrane 37. We speculated that sCD146 derived from BrM-CSCs may interact with VEGFR2 on ECs, as sCD146 retains the extracellular structural domain 38. Immunofluorescence experiments verified the co-localization between sCD146 and VEGFR2 and immunoprecipitation experiments confirmed that sCD146 binds to VEGFR2 of ECs (Figure 5E-F). Protein stability is an important factor that affects the abundance of receptor-type tyrosine kinase family proteins on the membrane 39. We therefore examined the protein stability of VEGFR2 in the presence of sCD146 and found that the protein stability of VEGFR2 was enhanced by sCD146 treatment (Figure 5G and S4F). Finally, the effect of sCD146 on the binding of VEGFA to VEGFR2 was explored through molecular dynamics (MD) simulations. The molecular alignment results showed that the domains of sCD146 bond to the contact surface between VEGFA and VEGFR2 (Figure 5H), indicating the possibility for sCD146 to regulate the interaction between VEGFA and VEGFR2. PCA results showed that the presence of sCD146 increased the tendency of two VEGFR monomers to move towards each other (Figure 5I), which resulted in an increase of binding free energy between VEGFA and VEGFR (Figure 5J). This suggests that binding of sCD146 stimulated VEGFR2 to be more sensitive to VEGFA by enhancing the interaction between VEGFR2 and VEGFA. Further *in vitro* assays confirmed that in the presence of sCD146, endothelial VEGFR2 underwent more activation in response to equal amounts of ligand stimulation (Figure 5K). Collectively, these data indicated that CD146 exerts a dual promotive effect on the VEGF-VEGFR axis by both up-regulating tumoral VEGFA transcription and stabilizing and sensitizing VEGFR2 on ECs as a co-receptor.

### Reactive astrocyte-derived GAS6 induces the overexpression of CD146 by AXL signaling

**Figure 6:**
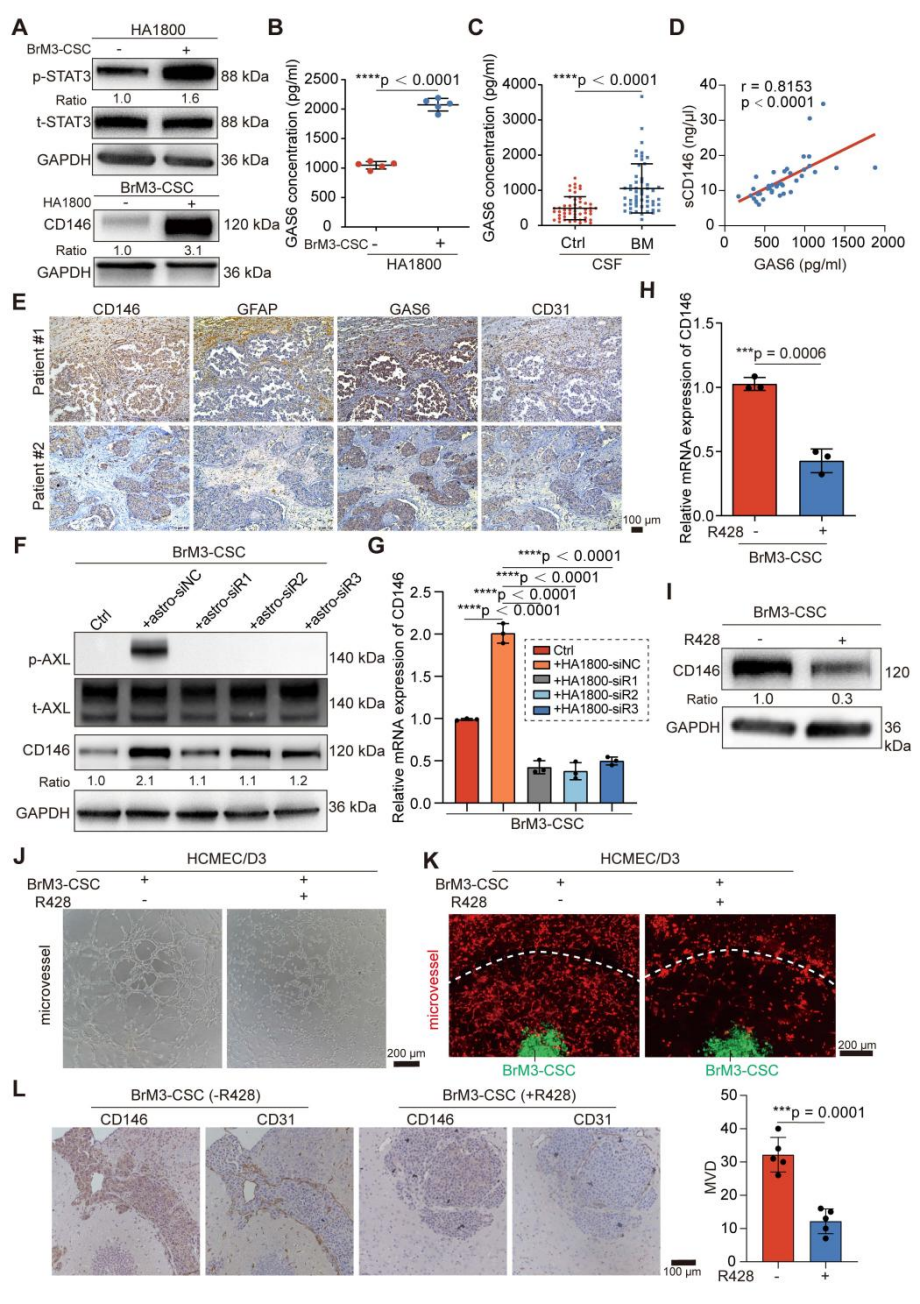
** Reactive astrocyte-derived GAS6 induces the overexpression of CD146 by AXL signaling.** (A) BrM3-CSCs were co-cultured with human astrocyte HA1800 for 12h by using Transwell inserts. Western blot analysis showing protein expressions of phosphorylated stat3 (p-STAT3) in human astrocyte HA1800 cells and expressions of CD146 in BrM3-CSCs. n = 3. (B) ELISA showed the levels of GAS6 in the secretions of astrocytes cultured with or without BrM3-CSCs. Student's* t* test (two-sided). n = 5. (C) ELISA results showed the level of GAS6 in the cerebrospinal fluid samples (CSF, n = 104) of participants. BrM, lung cancer brain metastasis, n = 53. Ctrl, patients with non-BrM disease as control group, n = 51. Mann Whitney test. (D) A scatter diagram is used to illustrate the correlation between GAS6 and sCD146 levels in CSF samples from lung cancer BrM patients. Two-sided Spearman's correlation analysis. n = 38. (E) Representative images of CD146, GFAP, GAS6 and CD31 staining in the serial sections of metastasized tumors from lung cancer BrM patients. (F) Immunoblots for CD146 and phosphorylated AXL (p-AXL) in BrM3-CSCs co-cultured with indicated astrocytes (with or without GAS6 silence). n = 3. (G) Results of qPCR showing mRNA level of CD146 in BrM3-CSCs co-cultured with indicated astrocytes (with or without GAS6 silence). One-way ANOVA test. n = 3. (H-I) The levels of CD146 were detected by qPCR and Western blot in BrM3-CSCs treated with or without R428 (1 μM for 24h). Student's *t* test (two-sided). n = 3. (J-K) Representative images of *in vitro* HCMEM/D3 tube formation assays (J) and angiogenesis assays on microfluidic chip (K) showing the ability of BrM3-CSCs treated with or without R428 to induce angiogenesis. Scar bar, 200 μm. (L) Nude mice were injected intracranially with BrM3-CSCs (n = 10) and treated with or without R428 (75mg/kg, once a day, intragastric administration) (n = 5 per group). Intracranial tumors were obtained and serial sections of tumor were stained with CD146 and CD31. And the micro-vascular densities (MVDs) were quantified and analyzed. Scar bar, 100μm. Student's *t* test (two-sided). n = 5. astro-siNC, HA1800 cells transfected with negative control siRNA. astro-siR1, HA1800 cells transfected with GAS6-targeted siRNA oligo 1. astro-siR2, HA1800 cells transfected with GAS6-targeted siRNA oligo 2. astro-siR3, HA1800 cells transfected with GAS6-targeted siRNA oligo 3. Error bars are defined as s.d.

Research has shown that the local TME supports the mesenchymal shift of CSCs 40. Recent studies characterizing the spatial landscape of NSCLS BrM by single-cell sequencing and spatial transcriptomics revealed that the TME in the BrM microenvironment undergoes extensive remodeling to create an immunosuppressive, fibrogenic, and high vascularized niche for BrM cells and is characterized by the accumulation of reactive astrocytes, M2 microglia and tumor-associated ECs 32, 41. Therefore, we explored whether these resident cells in “soils” may be involved in the mechanism by which BrM-CSCs acquire elevated CD146 expression. We co-cultured BrM-CSCs with human astrocytes (HA1800), microglia (HMC3), and brain ECs (HCMEC/D3) to simulate and obtain similar alterations in the brain TME. Only the reactive astrocytes, characterized by active STAT3 and GFAP 42, significantly elevated CD146 expression in BrM-CSCs (Figure 6A and S5A-B). CD44^+^ H1650-CSCs and CD44^+^ lung cancer patient-derived organoids also showed increased CD146 expression after co-culture with astrocytes (Figure S5C-D). To investigate the mediator of astrocyte-induced CD146 expression in BrM-CSCs, the supernatant from the co-culture system and from astrocytes cultured alone were collected for proteomic analysis (Figure S5E-G). Previous BrM protein profiling revealed that AXL, a member of the TAM (TYRO3, AXL, MER) receptor tyrosine kinase family implicated in oncogenesis and metastasis of many cancer types is elevated in BrM cells 43. It was also verified that AXL is increased in BrM-CSCs (Figure S5H). We found that growth arrest-specific protein 6 (GAS6), the high-affinity ligand of AXL, was significantly upregulated in the supernatant from reactive astrocytes (Figure 6B) and the CSF samples of NSCLC BrM patients (Figure 6C, Table S3). Moreover, we detected a significant positive correlation between the level of GAS6 and sCD146/CD146 in CSF (Figure 6D) and tissues (Figure S5I) of NSCLC BrM patients. Staining of brain metastases from NSCLC patients revealed more clustered reactive astrocytes, GAS6 infiltration, and accompanying dense vascularity in tissues with CD146 high expression compared to tissues with CD146 low expression (Figure 6E), and a similarity was observed in the intracranial tumors from mice implanted with BrM3-CSCs (Figure S5J). Immunoblotting showed that GAS6 expression was specifically derived from reactive astrocytes and inhibition of astrocyte activation by STAT3 inhibitor (Stattic) significantly suppressed GAS6 expression (Figure S5K-L). We further silenced GAS6 expression in astrocytes (Figure S5M) and co-cultured the astrocytes with BrM-CSCs. The results showed that silencing GAS6 abolished the induction of CD146 transcription and expression by astrocytes on BrM-CSCs and suppressed activation of the receptor AXL (Figure 6F-G).

Bemcentinib (R428/BGB324), a small molecule inhibitor of AXL, suppressed the transcription and expression of CD146 in BrM-CSCs and the ability to induce angiogenesis *in vitro* and in the *ex vivo* chip (Figure 6H-K and S5N-O). We also administered bemcentinib to mice that were intracranially inoculated with BrM-CSCs and found that bemcentinib significantly inhibited the growth and vascularization of brain tumors *in vivo* (Figure 6L and S5P), indicating that AXL signaling is indispensable for the overexpression of CD146 and its mediated angiogenesis. We further demonstrated that under the activation of AXL, the activator protein 1, a dimeric transcription factor consisting of c-Jun and c-Fos, accumulates more in the nucleus and binds to a highly conserved sequence (TGACGTCA) that is essential for gene transcription 44 in the CD146 promoter, thereby initiating activation of CD146 transcription (Figure S6A-H). In summary, these data indicated that reactive astrocyte-derived GAS6 induces the overexpression of CD146 by AXL signaling in BrM-CSCs.

### Imaprelimab and bemcentinib exhibit stronger anti-angiogenic effects than Bev *in vivo*

**Figure 7:**
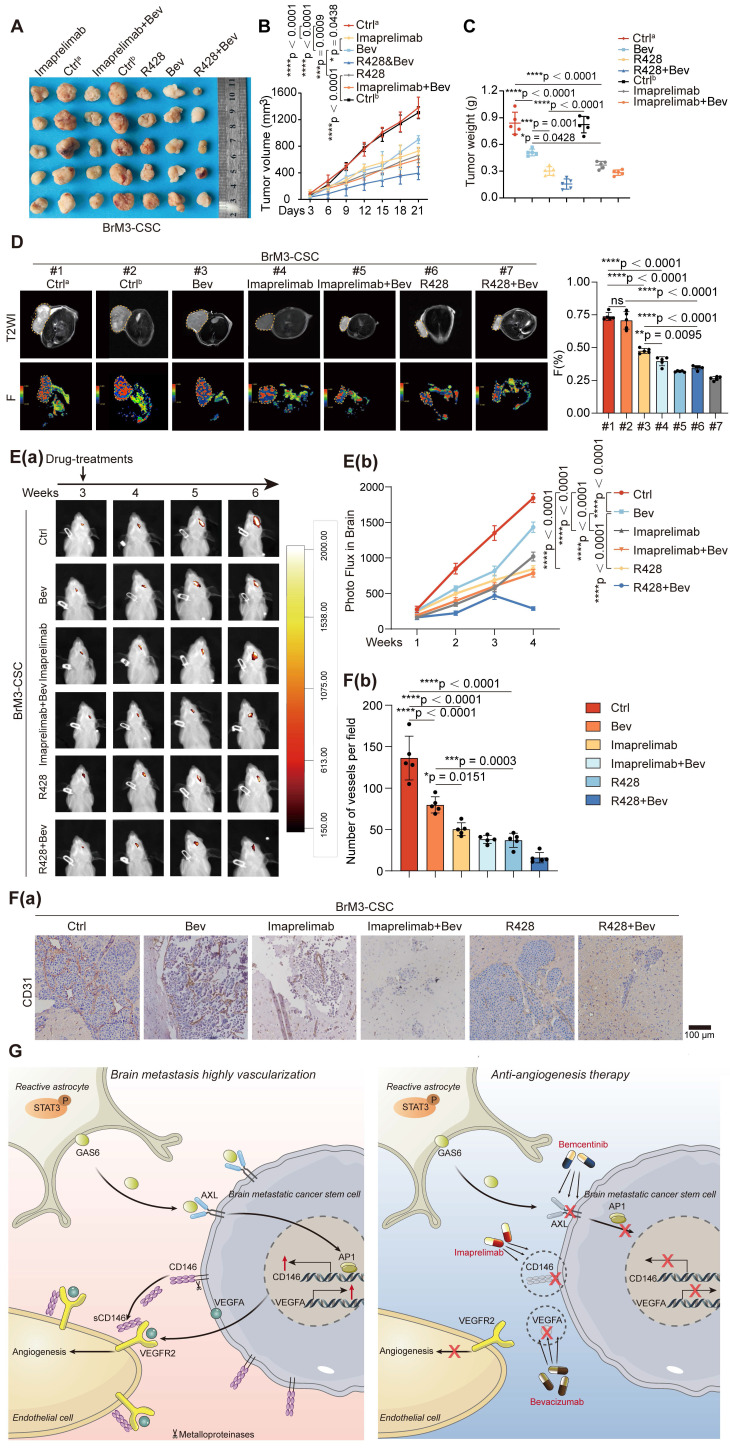
**Combination therapy of targeting-CD146 plus Bev exhibits greater anti-angiogenesis activity for BrM than Bev alone.** (A-D) Nude mice were subjected to subcutaneous injection with BrM3-CSCs, and treated with PBS as control^a^ (Ctrl^a^, twice a week, intraperitoneally (i.p), n = 5), or carboxymethylcellulose sodium (CMC-Na) as control^b^ (Ctrl^b^, once a day, intragastric administration (i.g.), n = 5) or Bev (5mg/kg, twice a week, i.p., n = 5), or bemcentinib (R428, 75mg/kg, once a day, i.g., n = 5), or imaprelimab (10 mg/kg, every two days, i.p., n = 5), or Bev and bemcentinib/imaprelimab combined (n = 5 per group). Tumor size measurements were taken every 3 days via a caliper to draw the growth curve (B) and animals were examined by a 3.0-T magnetic resonance imaging (MRI) system when the treatment had lasted for 21 days (D). Weighing of tumor mass was performed when the experiment concluded (A and C). (D) Representative MRI T2W imaging (T2WI) images and F map images for each group and quantification analysis of F value. n = 5. (E-F) Animals were injected intracranially with BrM3-CSCs, and treated with PBS as control (Ctrl, twice a week, i.p., n = 5), or Bev (5mg/kg, twice a week, i.p., n = 5), or bemcentinib (R428, 75mg/kg, once a day, i.g., n = 5), or imaprelimab (10 mg/kg, every two days, i.p., n = 5), or Bev and bemcentinib/imaprelimab combined (n = 5 per group). Brain colonization was analyzed by bioluminescence imaging (BLI) weekly and the treatments were started at the third week after injection. Heat map images of bioluminescence intensity for representative mice were presented (E(a)) and quantification analysis of bioluminescence readings was performed (E(b)). Animals were sacrificed and brain tissues were obtained after 3-weeks treatment. Sections of intracranial tumor of mice were stained with anti-CD31 antibody (F(a)), and the blood vessel densities were quantified and analyzed (F(b)). Scar bar, 100μm. n = 5. B, E, two-way ANOVA test. C, D, F, one-way ANOVA test. (G) Schematic diagram depicting the underlying mechanism of hypervascularization in NSCLC BrM that increased CD146 expression in brain metastatic cancer stem cells is induced by the reactive astrocytes-derived GAS6 and exerts a potent pro-angiogenesis role by the dual reinforcement on VEGF/VEGFR axis. Targeting CD146 by imaprelimab or bemcentinib significantly suppresses the angiogenesis and enhances the therapeutic effect of Bev, presenting a new anti-vascular strategy for lung cancer BrM. Bev, bevacizumab. Error bars are defined as s.d.

Using a subcutaneous Matrigel plug tumor model, we found that either imaprelimab (an antibody for CD146) or bemcentinib (R428) effectively inhibited subcutaneous tumor growth; furthermore, they showed a significantly increased effect compared with Bev, manifested by stronger suppression of subcutaneous tumor growth (Figure 7A-C). We used the pre-established diffusion-weighted imaging (DWI)-MRI and intravoxel incoherent motion (IVIM)-MRI imaging sequence parameters 15, 16, which can visualize tumor proliferation and angiogenesis *in vivo*, to assess the efficacies of the anti-vascular therapy in each group on day 21 of treatment. The results showed that the inhibition of tumor vascular density in imaprelimab and bemcentinib treatment group was greatly enhanced (Figure 7D) and the proliferation of tumor was accompanied by similar changes in vascular density (expressed as ADC and D-values) (Figure S7A).

The permeability of certain drugs into the brain is limited. Therefore, we further evaluated the effectiveness of these treatments in mice bearing intracranial tumors. Consistent with the results observed in subcutaneous tumors, either imaprelimab or bemcentinib could more significantly inhibited intracranial tumor growth than Bev (Figure 7E). Finally, staining of isolated subcutaneous and intracranial tumors showed that imaprelimab or bemcentinib reduced more vascular density than Bev (Figure 7F and S7B). Taken together, these results suggest that imaprelimab and bemcentinib exhibit stronger anti-angiogenic effects than Bev.

## Discussion

Brain tumors are highly angiogenic. VEGFA is the most potent pro-angiogenic factor and binds to VEGFR2 on the tumor endothelium 45. Bev, a widely used anti-VEGFA drug in clinical practice, has recently been found to improve survival for patients with BrM originating from lung cancer 46, 47. However, some patients are intrinsically refractory or acquired resistance to Bev. The mechanisms of resistance to Bev are diverse and include the following reasons: 1) the redundancy of other pro-angiogenic factors (e.g., basic fibroblast growth factors (bFGF), platelet-derived growth factor (PDGF)); 2) mesenchymal stromal cells and pathological stimuli in the TME activate and reinforce VEGFA production; and 3) VEGFR2 activation is sustained by other factors (e.g., programmed cell death-ligand 1 (PD-L1)). Many studies that were subsequently performed focused on the further elucidation of the tumor intrinsic or TME-related mechanisms of VEGFA regulation and release; clinical trials evaluating other anti-angiogenic factors drugs and development and testing studies to direct target VEGFR2 have also been pursued 48. However, these attempts have yielded limited results, especially in lung cancer BrM.

Here we found that CD146^+^ pericyte-like tumor cells derived from CD44^+^ BrM-CSCs potently exert pro-angiogenic effects through dual effects on the VEGFA-VEGFR2 axis in lung cancer BrM. This result is particularly promising given the above-mentioned obstacles in identifying successful VEGFA-targeting strategies. We found that the acquired high expression of CD146 regulates endogenous VEGFA production in brain metastatic cells. RNA-seq also revealed that CD146 upregulates a broad spectrum of pro-angiogenic factors, indicating that CD146 is a potent contributor to the hypervascularization of BrM. Furthermore, our results showed that its soluble form, sCD146, which was increased in the serum and CSF of lung cancer patients with BrM, directly binds to VEGFR2 on ECs, stabilizing and sensitizing VEGFR2 to its ligand as a co-receptor. These findings indicate that CD146, targeting both VEGFA and VEGFR, may represent an ideal anti-angiogenesis target. CD146 was originally thought to be a marker for pericytes. Recent studies showed CD146 or sCD146 is highly expressed in malignant tumors, and our work that highlights the mesenchymal transformation of CSCs gives a possible explanation for CD146 overexpression in tumors 49, 50. Notably, a study that developed a tumor-specific antibody against CD146, which recognizes CD146 expressed in cancer cells but not in pericytes, indicated the different structural features of tumor-derived CD146 from those of physiological CD146 51, implying the possibility of specifically targeting tumor CD146.

Considering the potential of CD146 as a therapeutic target for anti-angiogenesis therapy, neutralizing antibodies to CD146 (AA98 and imaprelimab) have been developed and exhibited efficacy in many preclinical trials for a wide range of diseases 52, 53. AA98 shows a significant anti-vascular effect in tumor-bearing mice 53; however, more research is needed before AA98 can be investigated in clinical trials. Imaprelimab, a CD146 antibody approved for clinical testing, was tested for safety, tolerability, pharmacokinetics and immunogenicity in approximately 40 healthy volunteers (ClinicalTrials.gov ID: NCT2458677) and 56 patients with psoriasis (ClinicalTrials.gov ID: NCT02630901). The outcomes of these two trials are not yet publicly available. Here we showed that imaprelimab effectively inhibits the vascularization of lung cancer BrM and significantly improves the efficacy of Bev in mice, suggesting that imaprelimab may be considered in clinical trials for anti-vascular treatment for lung cancer patients with BrM, if it shows qualified safety, tolerability, pharmacokinetics and immunogenicity in humans. In addition, considering the specificity of intracranial disease, assessing the permeability of imaprelimab to the blood-brain barrier in future studies is necessary.

To identify other strategies to effectively inhibit CD146, we explored the mechanisms by which CD146 is upregulated in BrM-CSCs. Recent studies found that CD146 is overexpressed in primary brain tumors (glioblastoma) and sCD146 mediated the resistance to anti-angiogenic therapy in glioblastoma 33, 54. Considering the similar brain microenvironment and hypervascularization characteristics in both primary and secondary brain tumors, we proposed that the specific brain TME microenvironment contributes to the overexpression of CD146. We found that reactive astrocytes are responsible for the increased CD146 expression in BrM-CSCs. The TME in brain tumors is characterized by the accumulation of reactive astrocytes and formation of carcinoma-astrocyte gap junctions, which are required for tumor colonization and development in the brain 55. As the most residential mesenchymal cells in brain TME, astrocytes undergo the transition from a naive to a metastasis-promoting reactive state and have emerged as a critical cell type that plays an important role in cerebral vascularization 56. Our results revealed a regulatory axis by which elevated GAS6 secreted by reactive astrocytes activates the receptor AXL on the surface of BrM-CSCs, which induces the transcription of CD146, highlighting the role of the TME in engaging the mesenchymal transformation of CTCs and tumor angiogenesis. A GAS6/AXL regulatory axis was recently reported between cancer-associated fibroblasts and cancer cells in gastric cancer, suggesting that GAS6/AXL is an important bridge for communication between the tumor and the TME 57. We also found that expression of the receptor AXL was upregulated in BrM cells, suggesting hyper-activated GAS6/AXL signaling in BrM; this should be explored in future studies.

Increasing studies have emphasized the important roles of GAS6/AXL in tumor progression and TME reprogramming in multiple malignancies including NSCLC 58, making it an emerging therapeutic target. Many AXL-targeted drugs have been developed including bemcemtinib (R428/BGB324). These agents, alone or in combination with other therapies, were shown to improve cancer patient prognosis in clinical trials 59. As GAS6/AXL was demonstrated to have a broad effect on the immune TME, which facilitates an immunosuppressive TME, bemcentinib (R428/BGB324) has been granted fast track designation by the U.S. Food and Drug Administration in STK11-mutated advanced metastatic NSCLC patients that have a suboptimal response to immune checkpoint blockade (NCT03184571) 60. A recent Phase 1 trial showed the anti-tumor activity of bemcentinib plus docetaxel in previously treated, advanced NSCLC 61. These findings indicate a great potential of bemcentinib for the treatment of advanced lung cancer. In this study, we explored the therapeutic effectiveness of bemcemtinib in a preclinical model of lung cancer BrM. Our results demonstrated that bemcemtinib exerts superior anti-vascular effects to Bev, which may be from its inhibition on the differentiation of CD146^+^ pericyte-like cells and the dual regulatory effect on VEGF and VEGFR, indicating that bemcemtinib may be a potent anti-VEGF/VEGFR agent. Additionally, stronger anti-angiogenesis effects were observed with the bemcemtinib and Bev combination. These findings provide evidence for the use of bemcemtinib in lung cancer patients with BrM and warrant further testing of its efficacy and tolerability in clinical trials.

## Conclusions

Here we demonstrated that BrM-CSCs mimic the pericytes by overexpressing CD146 in the brain TME, which is induced by the reactive astrocyte-derived GAS6, and exerts a potent pro-angiogenesis effect by dual effects on the VEGFA/VEGFR axis in lung cancer BrM. Imaprelimab and bemcentinib exhibit stronger anti-angiogenic effects than Bev *in vivo*, representing new anti-vascular strategies in NSCLC-BrM (Figure 7G).

## Supplementary Material

Supplementary figures and tables.

## Abbreviations

NSCLC: non-small cell lung carcinoma
BrM: brain metastasis
CSC: cancer stem cell
CD146: melanoma cell adhesion molecule
sCD146: soluble melanoma cell adhesion molecule
TME: tumor microenvironment
VEGF: vascular endothelial growth factor
VEGFR: vascular endothelial growth factor receptor
GAS6: growth arrest specific 6
EC: endothelial cell
Bev: bevacizumab
AXL: AXL receptor tyrosine kinase
ABCG2: ATP binding cassette subfamily G member 2
PDGFRβ: platelet derived growth factor receptor beta
NG2: new glue 2
p-VEGFR2: phosphorylated vascular endothelial growth factor receptor 2
ERK: extracellular signal-regulated kinase
MAPK: mitogen-activated protein kinase
STAT3: signal transducer and activator of transcription 3
INSR: insulin receptor
HSPG2: heparan sulfate proteoglycan 2
GFAP: glial fibrillary acidic protein
c-Jun: Jun proto-oncogene
c-Fos: fos proto-oncogene
AP-1: activator protien-1
GAPDH: glyceraldehyde phosphate dehydrogenase
STR: short tandem repeat
PBT: primary brain tumors
HG: healthy group
ELC: early lung cancer
LM: live metastasis
CSF: cerebrospinal fluid
IRS: immunoreactive score
OS: overall survival
MD: molecular dynamics
DWI: diffusion-weighted imaging
IVIM: intravoxel incoherent motion
MRI: magnetic resonance imaging
FGF: fibroblast growth factor
PDGF: platelet-derived growth factor
q-PCR: quantitative-polymerase chain reaction
RNA-seq: ribonucleic acid sequencing
IHC: immunohistochemistry
ELISA: enzyme-linked immunosorbent assay
ChIP: chromatin immunoprecipitation
