# Supplementary material for: CD146+ pericyte-like lung cancer brain metastatic stem cells promote tumor angiogenesis through dual regulatory effects on the VEGF/VEGFR axis
Source: Theranostics. 2026 Jan 1;16(4):1905–24. doi: 10.7150/thno.122241 (PMC12680595; doi:10.7150/thno.122241)
Supplement: Supplementary file 1 — Supplementary figures and tables. [file thnov16p1905s1.pdf]

Materials and methods

Endothelium conditioned medium collection

Indicated tumor cells were cultured in regular growth medium and allowed for 70%-80% confluency. The culture medium was then replaced for additional 24 h with incomplete medium without FBS. Thereafter, the supernatants were collected, centrifuged at 4000 rpm for 5 min to remove cell debris, and used as CM for HCMEC/D3 cells.

Endothelial tube formation, adhesion, proliferation, migration and invasion assays

Tube formation assays. Growth factor-reduced matrigel was plated on the bottom of pre-coated 96-well plates, and left at 37°C for 1 h for gelation. 20,000 starved HCMEC/D3 cells were resuspended in indicated conditioned medium (CM) from tumor cells, then seeded on Matrigel and incubated at 37°C. After 16 h, the HCMEC/D3 cells were imaged directly, or were then fixed, permeabilized and stained by SF488-labelled Phallotoxins (CA1640, Solarbio). The images were obtained under an inverted fluorescence microscope (ECLIPSE Ti-s, Nikon) and qualificated by AngiTool software.

Adhesion assays. HCMEC/D3 cells were seeded in 96-well plates and allowed to reach 100% confluence. 3×10^4 suspended GFP-tumor cells in 100 μL complete medium were seeded on endothelial single cell layer and left to stand for 10 min, then 100 μL serum-free medium was aspirated and blown 3 times, repeated 3 times and then imaged under an inverted fluorescence microscope (ECLIPSE Ti-s, Nikon).

Proliferation assays. 10^3 HCMEC/D3 cells were harvest and put into 6-well plates and allow to proliferate in indicated CM for 10 days. CM replaced every 24 h. The colonies that were obtained were fixed in 4% paraformaldehyde solution for a duration of 10 min, followed by staining with a 1% crystal violet solution for 20 min.

Migration assays. To assess the migration abilities of endothelial cells under indicated CM, HCMEC/D3 cells were cultured in 6-well plates in regular medium containing 10% FBS. Once the cells grew to 80-90% confluency, wounds were made using a sterile 100 μL pipette tip, washed up by phosphate buffer saline (PBS), and maintained in indicated CM. The wound areas were captured by an inverted microscope (Nikon TE200) at 0 and 24 h post wounding. The experiments were performed in triplicate.

Invasion assays. HCMEC/D3 cells were seeded into the top chamber of 24-well transwell plates.

These plates featured an 8-μm pore size (Corning, USA) and had their top chambers pre-coated with Matrigel. The seeded cells were maintained in the specified conditioned medium (CM), while the bottom chamber of each plate was filled with complete medium. Following a 24-hour incubation period, non-invasive cells on the upper surface of the top chamber were wiped off using a cotton swab. Invasive cells adhering to the bottom surface of the top chamber were then fixed with 4% paraformaldehyde and stained with 2% crystal violet. Subsequent imaging was conducted using a Leica TCSSP5II microscope. For quantification, invading cells were counted in five randomly chosen regions per image, with the counting performed in a blinded fashion.

Flow cytometry (FC)

Cells were detached using Accutase® Cell Detachment Solution (423302, BioLegend), and then were centrifuged and resuspended in Cell Staining Buffer. Cells were incubated with Human TruStain fcX™ (422301, BioLegend) for FC receptor blockage and then incubated with APC-anti-human CD309 (VEGFR2) Antibody (A16985H, BioLegend) for 45 min on ice away from light. Cell suspensions were washed with Cell Staining Buffer twice and analyzed by flow cytometry (CytoFlex LX, Beckman).

RNAi and plasmid design and transfection

The siRNA and plasmid for CD146, GAS6, cFos and cJun were designed and synthesized by YouBio (China). The target sequences of CD146, GAS6, cFos and cJun plasmids were referred to its genetic sequence in the PubMed (https://www.ncbi.nlm.nih.gov/gene). The sequences of siRNA oligos were as followings:

siRNAs for GAS6:

siR-1: 5'- CGAAGAAACUCAAGAAGCATT -3' (sense)

5'- UGCUUCUUGAGUUUCUUCGTT -3' (anti-sense)

siR-2: 5'- GGAACUGGCUGAACGGAGATT -3' (sense)

5'- UCUCCGUUCAGCCAGUUCCTT -3' (anti-sense)

siRNAs for c-Jun:

siR-1: 5'- GGAUCAAGGCGGAGAGGAATT -3' (sense)

5'- UUCCUCUCCGCCUUGAUCCTT -3' (anti-sense)

siR-2: 5'- GAAGCGCAUGAGGAACCGCAUTT -3' (sense)

5'- AUGCGGUUCCUCAUGCGCUUCTT -3' (anti-sense)

siRNAs for c-Fos:

siR-1: 5'- AAAGGGUUCAGCCUUCAGCUCCAUG -3' (sense)

5'- CAUGGAGCUGAAGCCUGAACCCUUU -3' (anti-sense)

siR-2: 5'- AGGAGAAUCCGAAGGGAAATT -3' (sense)

5'- UUUCCCUUCGGAUUCUCCUTT -3' (anti-sense)

The siRNA oligos and plasmids were transfected by Lipofectamine 2000 (Invitrogen, Carlsbad, CA) following the manufacturer's instructions.

The CD146-targeting shRNAs and its negative control shRNA (shNC), and CD146 plasmid constructed in the LV2 lentiviral vector were packaged in 293T cells (GenePharma, Suzhou, China). The target sequences of CD146 shRNA1 and shRNA2 were 5′-GCCUUUGCCGCCUACUGAATT-3′, and 5′-GGAGUAACGAAGAGAUCAATT-3′, respectively. The target sequences of GSTM1 shRNA1 and shRNA2 were 5′-GAGCGAACTTGTAGTTGAA-3′, and 5′-GAGTGAACCACAGGAACTA-3′, respectively. Infection of the shRNA-expressing lentivirus and establishment of stably infected single clones were carried out as directed by the manufacturer. The knockdown efficiency was evaluated by western blot analysis.

Dual-luciferase reporter assays

The luciferase reporter constructs containing the human CD146 promoter fragments were established using the pGL3-basic vector by YouBio (China). 293T cells were transfected with the luciferase reporter constructs, cJun/cFos pcDNA3.1 plasmid and Renilla luciferase reporter vector, and the luciferase activity was detected using the Dual-Glo® Luciferase Assay Kit (E1910, Promega) with a microplate reader (Varioskan LUX, Thermo).

Chromatin immunoprecipitation (ChIP)

ChIP assays were performed using the ChIP kit (53040, Active Motif). The PCR primers were as follows: forward, 5′-TTGGCTCTCGCCCTCCG-3′, reverse, 5′-ATTTTTAGGCACGCTCCACCGCAG-3′.

Figures and legends

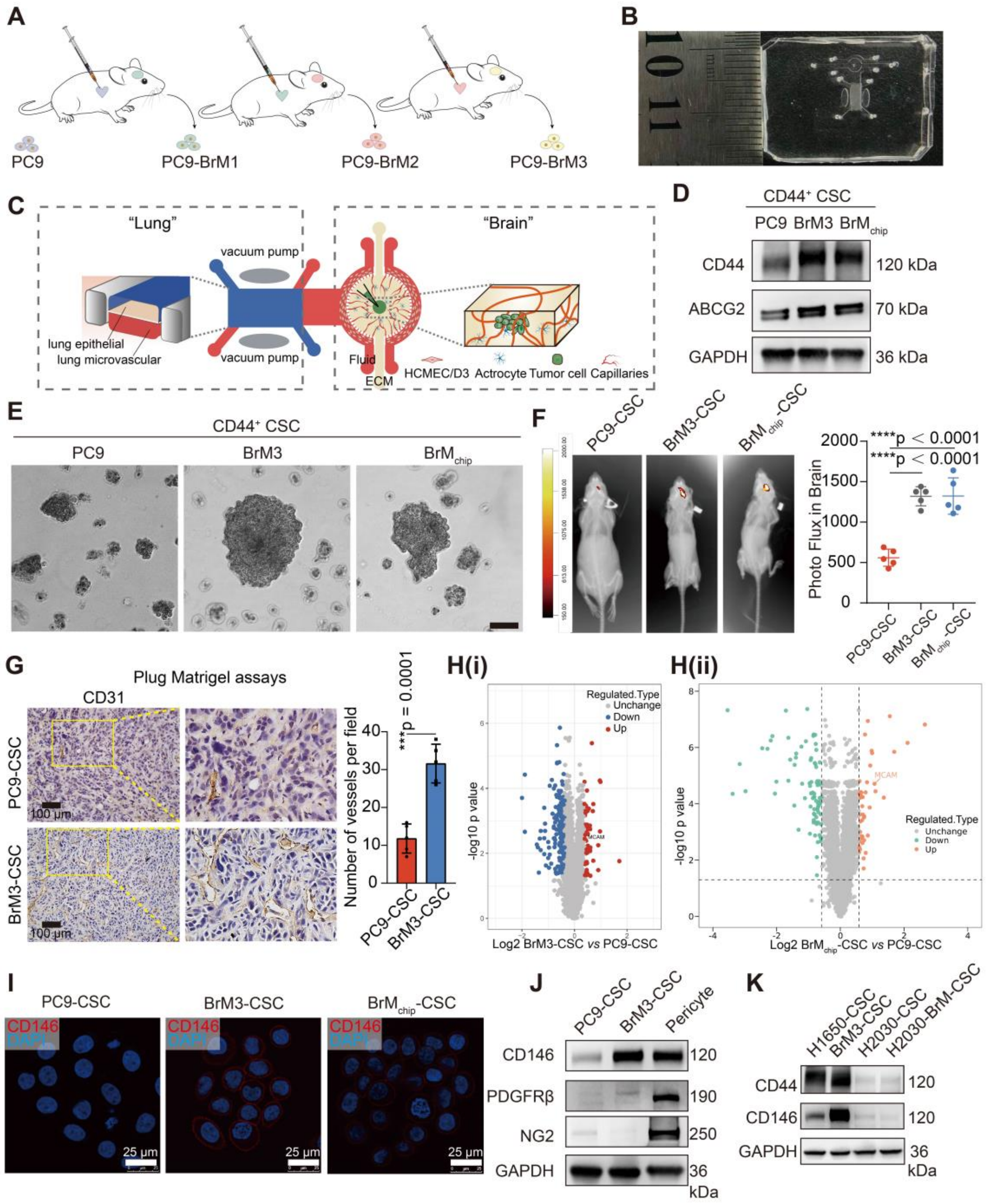

Figure S1. CD44^{+} lung cancer brain metastatic stem cells mimic pericytes through the acquired overexpression of CD146. (A) Schematic illustration of the *in vivo* isolation of metastatic populations (BrM1, BrM2, and BrM3) from the PC9 lung adenocarcinoma line. (B) The image of an actual BrM microfluidic chip as viewed from above. (C) Schematic illustration of brain metastases formation and subsequent angiogenesis on the BrM microfluidic chip. (D) Immunoblots for CD44 and ABCG2 in PC9-CSCs, BrM3-CSCs and BrM$_{chip}$-CSCs. (E) Representative morphology images of tumor spheres formed by PC9-CSCs, BrM3-CSCs and BrM$_{chip}$-CSCs. Scar

bar, 100μm. (F) Representative heat map image representations of bioluminescence intensity for mice intracranially injected with PC9-CSCs, BrM3-CSCs or BrM_{chip}-CSCs at the 4th week and the plot of mean bioluminescence readings. One-way ANOVA test. n=5. (G) Indicated tumor cells were injected into mice subcutaneously and the subcutaneous tumors were excised after 1 month. Images and quantification analysis of vessels in subcutaneous tumors by CD31 staining. Scar bar, 50μm. Student's *t* test (two-sided). n=5. (H) Volcano plots showed the upregulation of CD146 (MCAM) in BrM3-CSCs (i) and BrM_{chip}-CSCs (ii), compared with the parental PC9-CSCs. (I) Representative confocal images of CD146 immunofluorescent staining in indicated cells. Scar bar, 25μm. (J) Immunoblots for CD146, PDGFRβ and NG2 in PC9-CSCs, BrM3-CSCs and pericytes. (K) Immunoblots for CD44 and CD146 in indicated lung cancer cells. Error bars are defined as s.d.

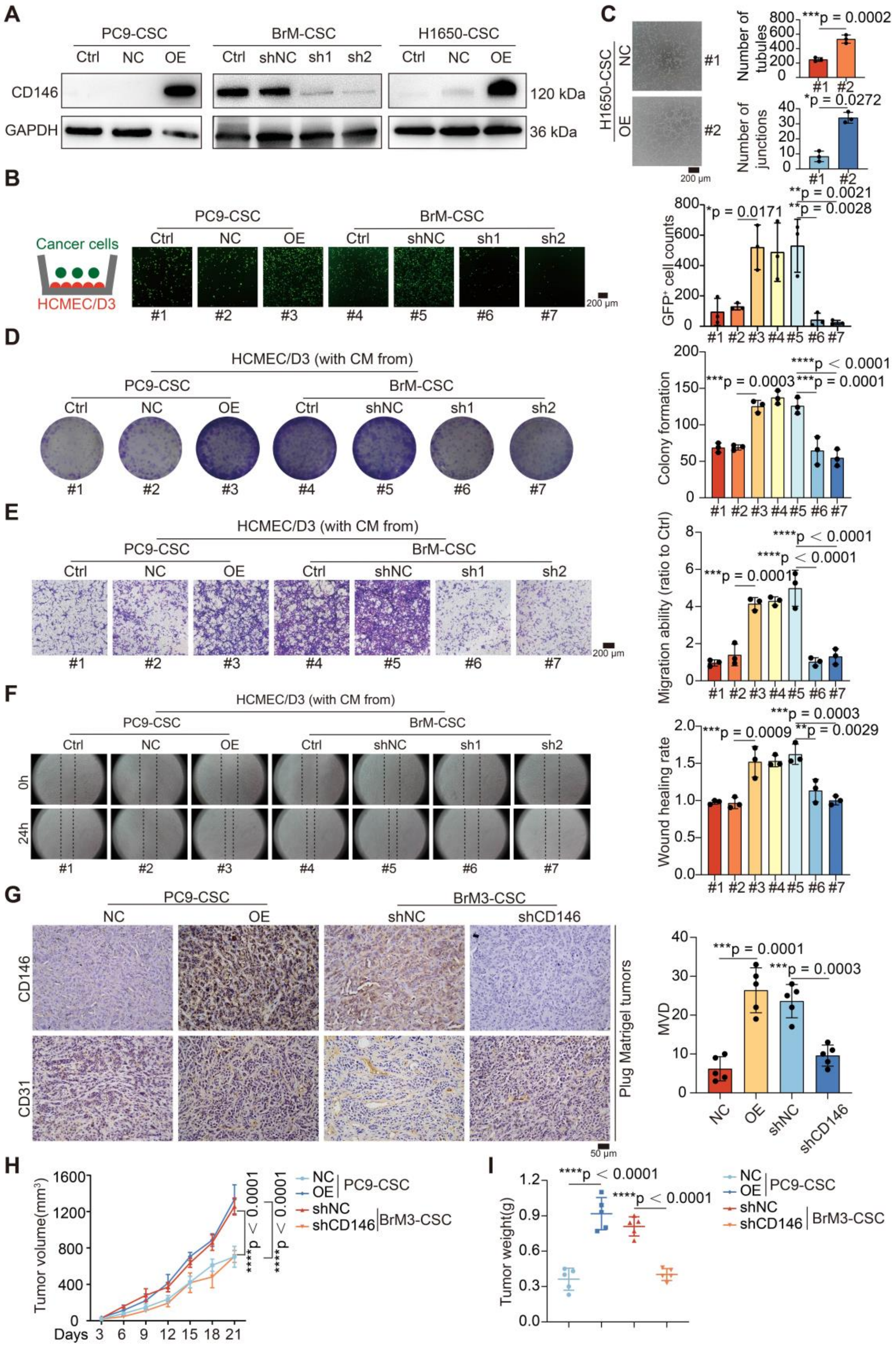

Figure S2. BrM-CSCs promote angiogenesis of BrM via CD146. (A) Western blotting showed the transfection efficacy in PC9 cells with CD146 overexpression (n=4) and PC9-BrM3 cells with

CD14 knock down (n=3). One-way ANOVA test. (B) Representative images and quantification analysis of the endothelial adhesion capacities of indicated tumor cells. Scar bar, 200μm. One-way ANOVA test. n=3. (C-E) Representative images and quantification analysis of the proliferation abilities of HCMEC/D3 cells by colony formation assays (C), the invasive abilities of HCMEC/D3 cells by Transwell invasion assays (D) and the migration abilities of HCMEC/D3 cells by wound healing assays (E), with the treatment of conditioned culture medium (CM) derived from indicated tumor cells. Scar bar, 200μm. One-way ANOVA test. n=3. (F) Subcutaneous Plug Matrigel tumors were obtained and stained with CD146 and CD31, and the micro-vascular densities (MVDs) were quantified and analyzed. Scar bar, 50μm. Student's t test (two-sided). n=5. (G) Subcutaneous tumor size was measured every 3 days using caliper to the plot growth curve. Student's t test (two-sided). n=5. (H) Subcutaneous tumor mass was weighed at the end of the experiment. Student's t test (two-sided). n=5. NC, PC9-CSCs transfected with negative control plasmid. OE, PC9-CSCs transfected with CD146 plasmid. shNC, BrM3-CSCs transfected with negative control shRNA. sh1, BrM3-CSCs transfected with CD146-targeted shRNA vector 1. sh2, BrM3-CSCs transfected with CD146-targeted shRNA vector 2. Error bars are defined as s.d.

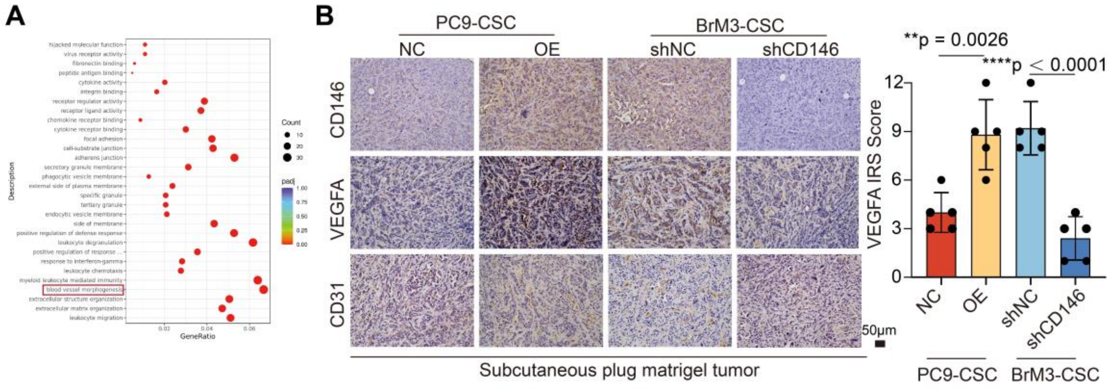

Figure S3. CD146 upregulates VEGFA in NSCLC BrM-CSCs. (A) Gene Ontology (GO) enrichment analysis of up-regulated differential genes in RNA-seq. (B) Representative images of CD146, VEGFA and CD31 staining in the serial sections of subcutaneous plug matrigel tumor of mice inoculated with indicated cells, and the IRS scores of VEGFA staining were quantified and analyzed. Scar bar, 100μm. n=3. Student's *t* test (two-sided). NC, PC9-CSCs transfected with negative control plasmid. OE, PC9-CSCs transfected with CD146 plasmid. shNC, BrM3-CSCs transfected with negative control shRNA. shCD146, BrM3-CSCs transfected with CD146-targeted

shRNA vector. Error bars are defined as s.d.

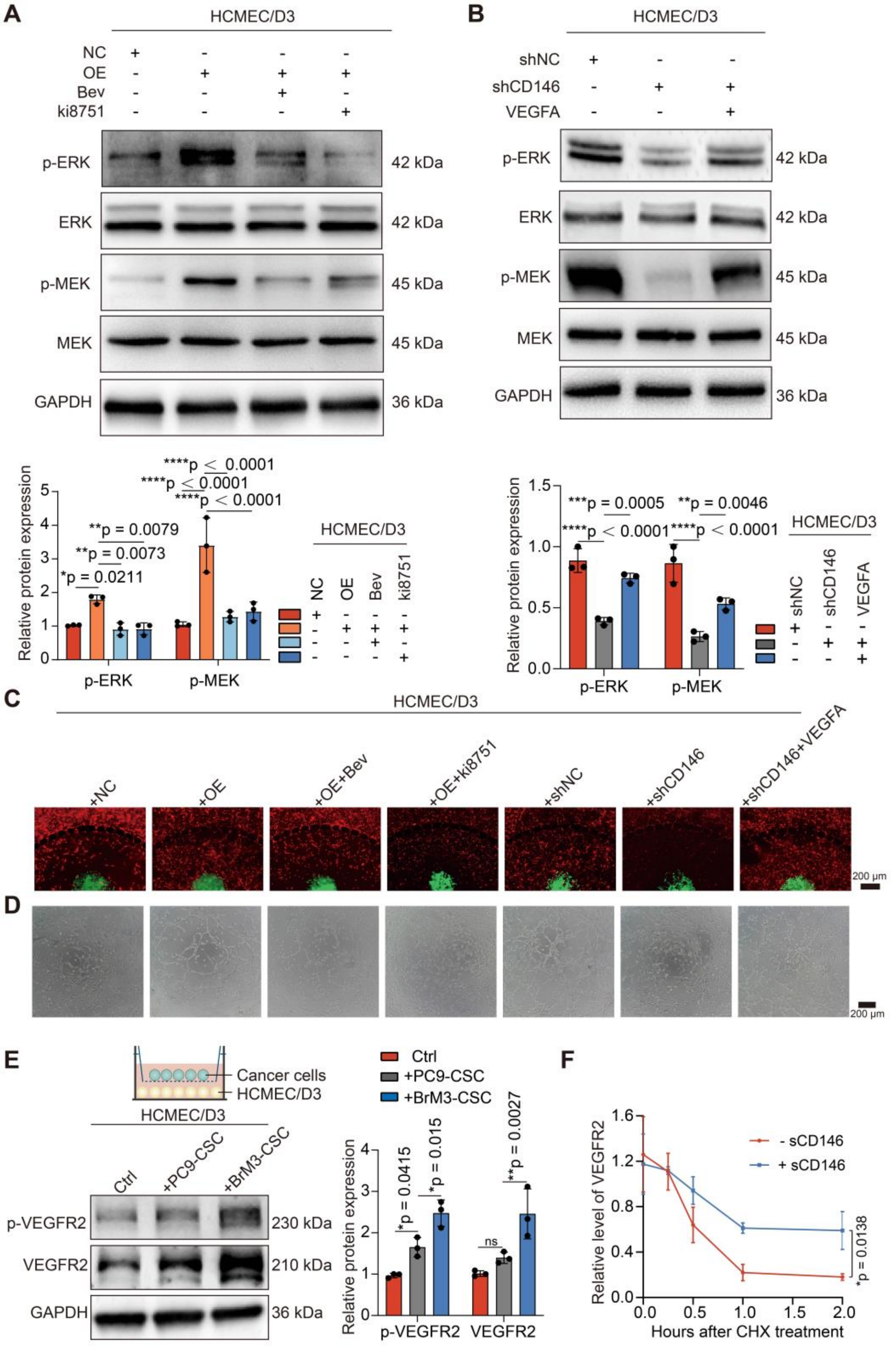

Figure S4. sCD146 amplifies and sensitizes VEGFR2 on the surface of cerebral microvascular endothelial cell. (A-B) Results of Western blot analysis showing protein expressions of MEK, ERK, phosphorylated MEK (p-MEK) and phosphorylated ERK (p-ERK) of HCMEC/D3 cells co-cultured

with indicated tumor cells with or without Bevacizumab (Bev, 100ng/ml), Ki8751 (1nM) or VEGFA (50ng/ml) treatment in a Transwell insert. Two-way ANOVA test. n=3. (C) Angiogenesis induced by indicated tumor cells and drug treatment on the BrM microfluidic chip. Scar bar, 200μm. n=3. (D) *In vitro* tube formation assays of HCMEC/D3 cells with the treatment of conditioned culture medium (CM) derived from indicated tumor cells combining indicated drugs. Scar bar, 200μm. n=3. (E) Western blotting assays showed the VEGFR2 and phosphorylated VEGFR2 (p-VEGFR2) level of HCMEC/D3 cells co-cultured with indicated tumor cells in a Transwell insert. Two-way ANOVA test. n=3. (F) Results of Western blot analysis showing protein expressions of VEGFR2 in individual groups of cells following treatment with CHX (50μM). Student's *t* test (two-sided). n=3. NC, PC9-CSCs transfected with negative control plasmid. OE, PC9-CSCs transfected with CD146 plasmid. shNC, BrM3-CSCs transfected with negative control shRNA. shCD146, BrM3-CSCs transfected with CD146-targeted shRNA vector. Error bars are defined as s.d.

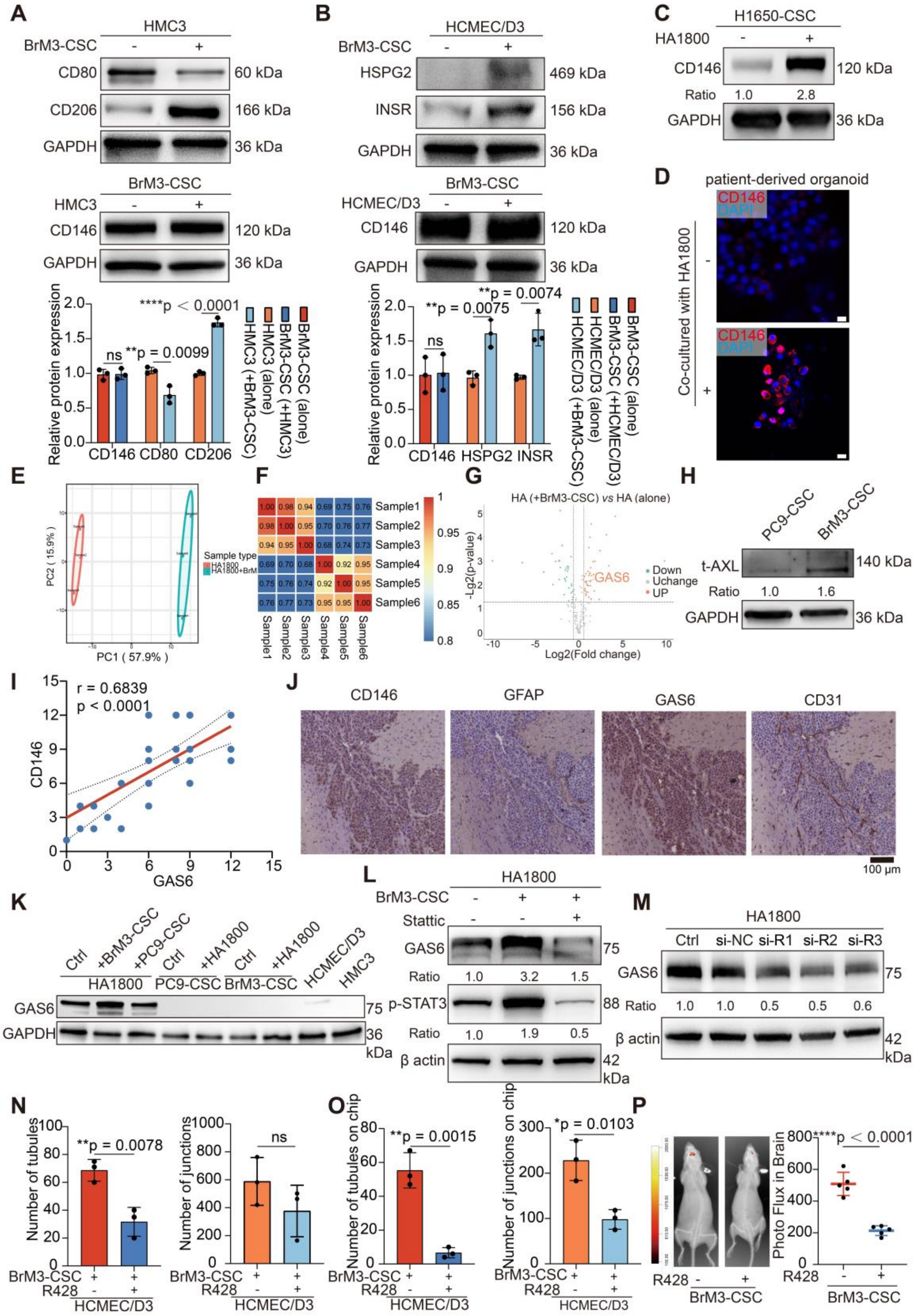

Figure S5. Reactive astrocyte-derived GAS6 induces the overexpression of CD146 by AXL signaling. (A) BrM3-CSCs were co-cultured with human microglia HMC3 for 48h by using Transwell inserts. Results of Western blot analysis showing protein expressions of CD80 and CD206

in human microglia HMC3 cells and expressions of CD146 in BrM3-CSCs. Student's *t* test (two-sided). n=3. (B) BrM3-CSCs were co-cultured with HCMEC/D3 cells for 24h by using Transwell inserts. Results of Western blot analysis showing protein expressions of HSPG2 and INSR in HCMEC/D3 cells and expressions of CD146 in BrM3-CSCs. Student's *t* test (two-sided). n=3. (C) H1650-CSCs were co-cultured with HA1800 for 12h by using Transwell inserts. Results of Western blot analysis showing protein expressions of CD146 in H1650-CSCs. Student's *t* test (two-sided). n=3. (D) Immunostaining of CD146 in NSCLC patient-derived tumor organoids with or without co-culture with astrocyte HA1800 for 12h by using Transwell inserts. Scar bar, 25μm. n=3. (E-F) Pearson's correlation coefficient (PCC) analysis (E) and principal component analysis (PCA) (F) indicating the good repeatability of samples for proteomics. (G) Volcano plot showed the upregulation of GAS6 in the secretions of reactive astrocytes co-cultured with BrM3-CSCs, compared with that of astrocytes cultured alone. (H) Results of Western blot analysis showing protein expressions of total AXL (t-AXL) in PC9-CSCs and BrM3-CSCs. n=3. (I) Scatter diagram showing the correlation between the GAS6 staining scores and the CD146 staining scores in the serial sections of metastasized tumors from lung cancer BrM patients. Pearson correlation analysis. n=35. (J) Representative images of CD146, GFAP, GAS6 and CD31 staining in the serial sections of the intracranial tumor from mice implanted with BrM3-CSCs. Scar bar, 100μm. (K) Immunoblots for GAS6 in indicated cells. n=3. (L) Human astrocyte HA1800 were co-cultured with BrM3-CSCs for 12h and administrated with Stattic (10μM for 12h) by using Transwell inserts. Immunoblots for GAS6 and phosphorylated stat3 (p-STAT3) in human astrocyte HA1800. n=3. (M) Immunoblots for GAS6 in HA1800 cells transfected with GAS6-targeted siRNA oligos. n=3. (N-O) *In vitro* HCMEM/D3 tube formation assays (N) and angiogenesis assays on microfluidic chip (O) showing the ability of BrM3-CSCs treated with or without R428 to induce angiogenesis. Student's *t* test (two-sided). n=3. (P) Heat map image representations of bioluminescence intensity for representative mice injected intracranially with BrM3-CSCs (n=6) and treated with or without R428 (75mg/kg, once a day, intragastric administration) (n=3 per group), and quantification analysis of bioluminescence readings. Student's *t* test (two-sided). Ctrl, control group. si-NC, transfection with negative control siRNA. Si-R1, transfection with GAS6-targeted siRNA oligo 1.si-R2, transfection with GAS6-targeted siRNA oligo 2. si-R3, transfection with GAS6-targeted siRNA oligo 3. Error bars are defined as s.d.

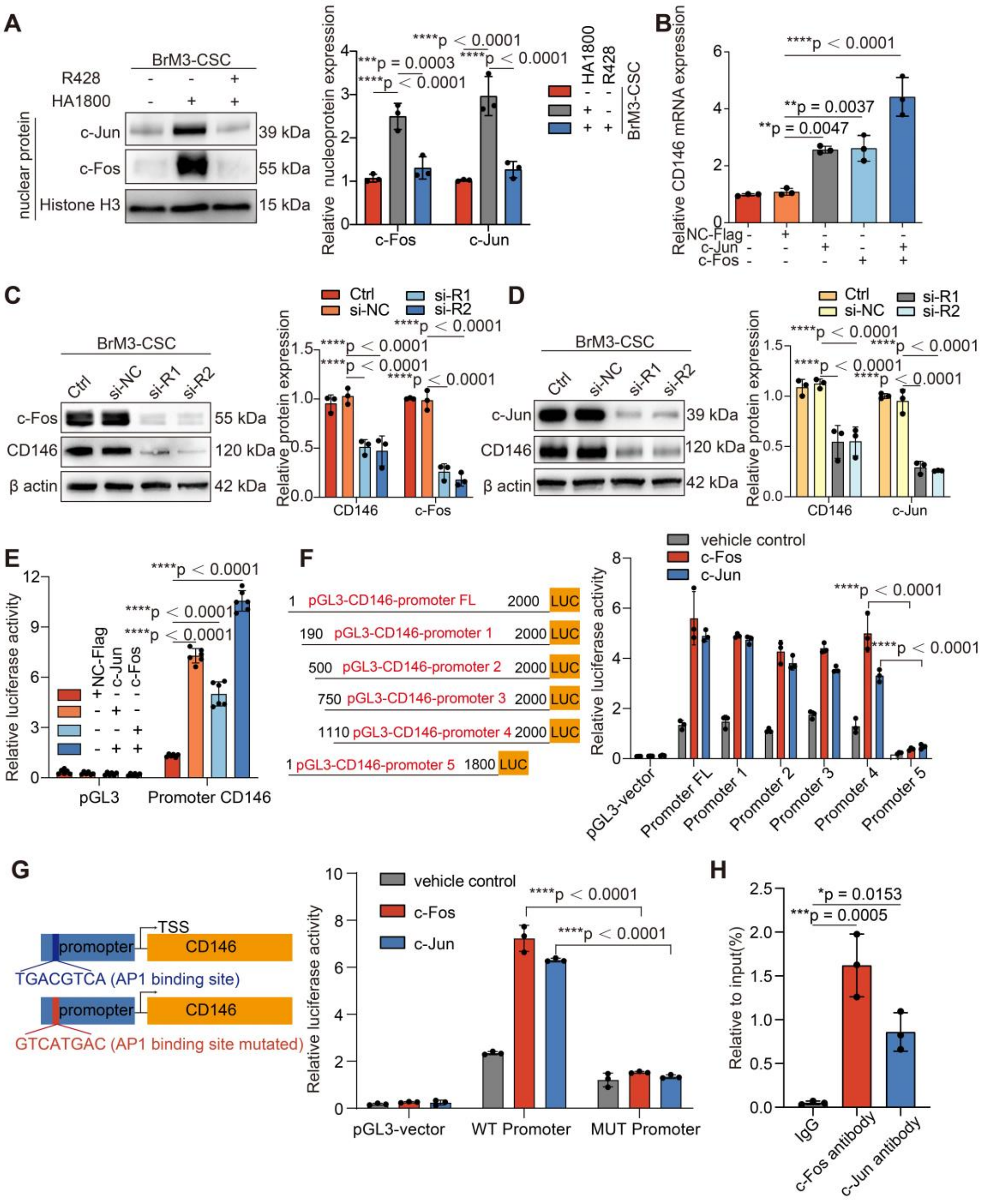

Figure S6. AP-1 activated by AXL signaling is responsible for the transcription of CD146 in BrM. (A) Results of Western blot analysis showing the nuclear c-Jun and c-Fos expression in BrM3-CSCs, BrM3-CSCs co-cultured with HA1800 cells, BrM3-CSCs co-cultured with HA1800 cells and treated with R428 (1μM). Histone H3 acted as the internal reference protein for nuclear protein. Two-way ANOVA test. n=3. (B) Results of qPCR analysis showing the mRNA level of CD146 in PC9-CSCs with cJun or/and cFos plasmid transfection for 48h. One-way ANOVA test. n=3. (C-D) Results of Western blot analysis showing the protein expression of CD146 in BrM3-CSCs with c-

Fos (C) or c-Jun (D) silence by using siRNA oligos. Two-way ANOVA test. n=3. (E) 293T cells were transiently transfected with the indicated pGL3 basic-based reporter constructs and c-Fos/c-Jun plasmid for 24 hours and then the luciferase activity was measured. Two-way ANOVA test. n=6. (F) Schematic illustration of a series of incremental deletion structures of the luciferase reporter gene (left) and the results of luciferase assays (right) where 293T cells were transiently transfected with the indicated pGL3 basic-based reporter constructs and c-Fos/c-Jun plasmid for 24 hours and then the luciferase activity was measured. Two-way ANOVA test. n=3. (G) Schematic illustration of the specific AP-1 binding sites of CD146 promotor (WT promoter) and the mutant sites (MUT promoter) for dual-luciferase assays (left). Results of luciferase assays (right): 293T cells were transiently transfected with the indicated pGL3 basic-based reporter constructs and c-Fos/c-Jun plasmid for 24 hours and then the luciferase activity was measured. TSS, transcription start site. One-way ANOVA test. n=3. (H) ChIP assays of AP-1 (c-Fos/c-Jun) binding to the CD146 promoter. n=3. One-way ANOVA test. NC, negative control. si-NC, transfection with negative control siRNA. Si-R1, transfection with siRNA oligo 1. si-R2, transfection with siRNA oligo 2. FL, full length. Error bars are defined as s.d.

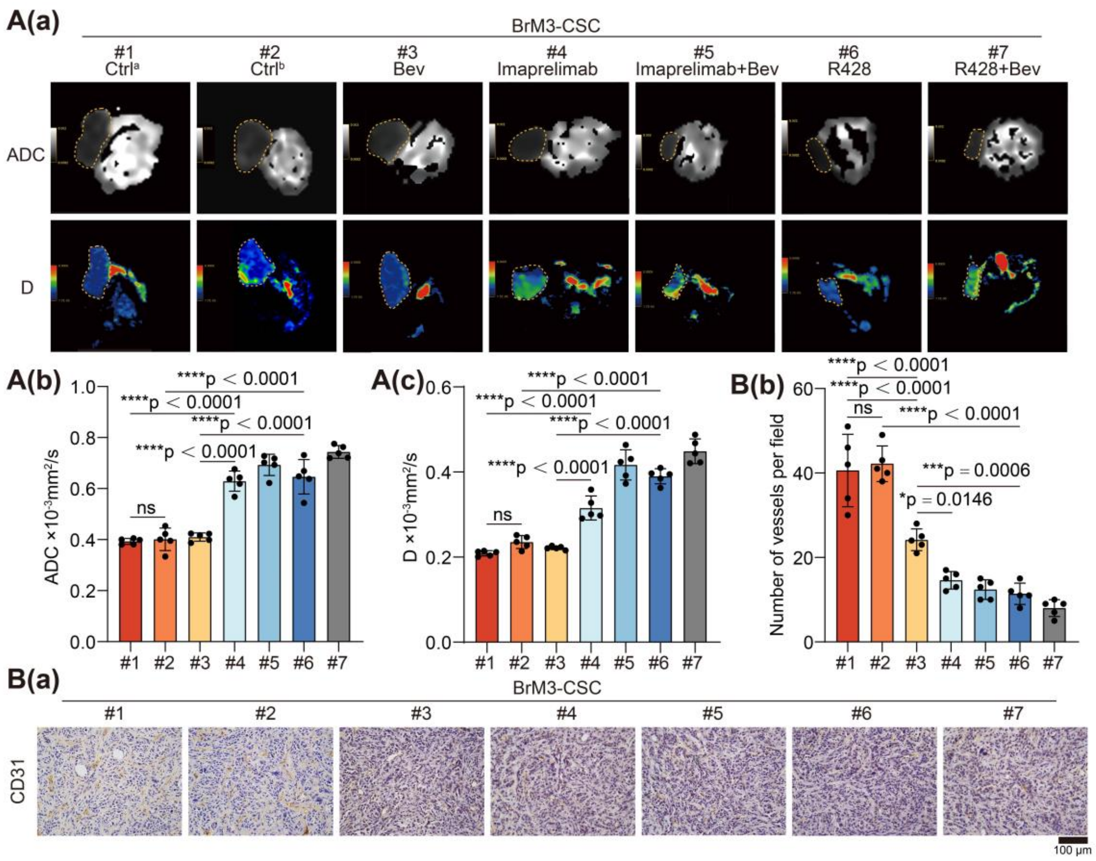

Figure S7. Combination therapy of targeting CD146 plus Bev exhibits greater anti-angiogenesis activity for BrM than Bev alone. Nude mice were injected subcutaneously with BrM3-CSCs, and treated with PBS as control[a] (Ctrl[a], twice a week, intraperitoneally (i.p), n=5), or Bev (5mg/kg, twice a week, i.p., n=5), or Bemcentinib (R428, 75mg/kg, once a day, intragastric administration (i.g.), n=5), or Imaprelimab (10 mg/kg, every two days, i.p., n=5), or Bev and Bemcentinib/Imaprelimab combined (n=5 per group). Animals were examined by a 3.0-T magnetic resonance imaging (MRI) system on the 21st day of treatment. (A) Representative ADC map and D map images (a) for each group and quantification analysis of values (b-c). One-way ANOVA test. n=5. (B) Sections of subcutaneous tumor of mice were stained with anti-CD31 antibody (a), and the blood vessel densities were quantified and analyzed (b). Scar bar, 100μm. One-way ANOVA test. n=5. Error bars are defined as s.d.

Table S1. **Correlation between CD146 expression and clinicopathological parameters in patients with lung cancer.**

Clinicopathological parameter	Variables	CD146 expression		Total	χ2	*p* value
		Low	High			
Age					1.115	0.291
	< 60	14	12	26		
	≥60	21	30	51		
Gender					0.312	0.577
	Female	12	17	29		
	Male	23	25	48		
Smoke					0.330	0.566
	Without	26	28	54		
	With	9	13	22		
	Null	0	1	1		
Histology					1.711	0.191
	Adenocarcinoma	32	34	66		
	Squamous	3	8	11		
T stage					0.456	0.499
	T1/T2	32	40	72		
	T3/T4	3	2	5		
N stage					0.369	0.544
	N0	9	9	18		
	N1/N2/N3	20	28	48		
	Null	6	5	11		
M stage					13.216	$<0.001^{***}$
	M0	27	15	42		
	M1	8	27	35		
	Null					
TNM stage					6.684	$<0.01^{*}$
	I/II	22	14	36		
	III/IV	13	28	41		

$^{*}p<0.05$, $^{***}p<0.001$

Table S2. **Clinicopathologic imaging features of the serum cohort.**

Characteristics	Case	Characteristics	Case
Lung cancer brain metastasis	44	Controls	116
Gender		Gender	
Male	20	Male	57
Female	24	Female	59
Age (Year)		Age (Year)	
≤60	22	≤60	69
>60	22	>60	47
Other organ metastasis		Type	
Without	24	NSCLC	69
With	20	Glioma	4
		Healthy group	24
		Acoustic Neuroma	1
		Meningeoma	8
		Brain lymphoma	2
		Pituitary tumour	7
		Spinal meningioma	1
Pathological type		Pathological type	
Adenocarcinoma	36	Adenocarcinoma	61
Squamous cell carcinoma	7	Squamous cell carcinoma	7
Other types	1	Other types	1
Number of brain metastatic lesions		TNM stage of NSCLC	
1	23	I / II	22
≥2	21	III / IV	47
Maximum diameter of BM (cm)		Organ metastasis of NSCLC	
≤2	31	Liver	22
>2	13	Bone	25
Meningeal metastasis		M stage of NSCLC	
Without	35	M0	22
With	9	M1	47
T stage of BM		T stage of NSCLC	
T1	5	T1	30
T2	14	T2	13
T3	9	T3	9
T4	16	T4	17
N stage of BM		N stage of NSCLC	
N0	6	N0	29
N1	1	N1	7
N2	18	N2	21
N3	19	N3	12

Table S3 Clinicopathologic features of the CSF validated cohort.

(i) CSF cohort for CD146 detection

Characteristics	Case	Characteristics	Case
**Lung cancer brain metastasis**	38	**Controls**	32
**Gender**		**Gender**	
Male	22	Male	16
Female	16	Female	16
**Age**		**Age**	
≤ 60	18	≤ 60	20
> 60	20	> 60	12
**Pathology**		**Diagnosis**	
Adenocarcinoma	33	Diffuse large B-cell lymphoma	1
Squamous carcinoma	5	Acute B lymphocytic leukemia	1
		Multiple Lacunar Infarcts	1
		Meningeoma	2
		Cerebral hemorrhage	6
		Hypophysoma	3
		Arteriovenous malformation	1
		Epilepsy	2
		Mycoplasma meningitis	1
		Encephalitis	2
		Glossopharyngeal neurinoma	1
		Cerebral infarction	1
		Glioma	1
		Myodystony	1
		Acoustic neurilemoma	1
		Psychobehavioral abnormality	1
		Spinal cord demyelinating disease	1
		Spastic paraplegia	1
		Arterial aneurysm	1
		Guillain-barre syndrome	1
		Breast cancer brain metastases	1
		Glioblastoma	1

(ii) CSF cohort for GAS6 detection

Characteristics	Case	Characteristics	Case
**Lung cancer brain metastasis**	51	**Controls**	53
**Gender**		**Gender**	
Male	26	Male	27
Female	25	Female	26
**Age**		**Age**	
≤ 60	24	≤ 60	31
> 60	27	> 60	22
**Pathology**		**Diagnosis**	
Adenocarcinoma	46	Diffuse large B-cell lymphoma	6
Squamous carcinoma	5	Acute B lymphocytic leukemia	3
		Fungal meningitis	1
		Lacunar infarctio	1
		Meningeoma	3
		Cerebral hemorrhage	8
		Viral encephalitis	2
		Intracranial hemangioma	1
		Hypophysoma	5
		Cerebral hernia	1
		Arteriovenous malformation	1
		Epilepsy	2
		Mycoplasma meningitis	1
		Encephalitis	2
		Glossopharyngeal neurinoma	1
		Cerebral infarction	1
		Glioma	1
		Myodystony	1
		Acoustic neurilemoma	1
		Psychobehavioral abnormality	1
		Spinal cord demyelinating disease	1
		Spastic paraplegia	1
		Arterial aneurysm	1
		Guillain-barre syndrome	1
		Breast cancer brain metastases	1
		Glioblastoma	2
		Rathke's cleft cyst	1
		Anterior communicating aneurysm	1
		Multiple Lacunar Infarcts	1

Table S4. **Correlation analysis of CD146 expression and clinicopathological imaging features in serum validated cohort.**

Characteristics	Case	CD146_*P*	Characteristics	Case	CD146_*P*
Gender			Number of lung primary lesions		
Male	20 (45.45%)	0.225	1	18 (40.90%)	0.352
Female	24(54.55%)		≥2	26 (59.10%)	
Age (Year)			Maximum diameter of LP (cm)		
≤60	22 (50%)	0.842	≤2	17 (38.64%)	0.571
>60	22 (50%)		>2	27 (61.36%)	
Pathological type			Pleural effusion		
Adenocarcinoma	36 (81.81%)		Without	26 (59.10%)	
Squamous cell carcinoma	7(15.91%)	0.962	With	18 (40.90%)	0.527
Other types	1 (2.28%)		Lung surgery		
Smoke			Without	32 (72.73%)	0.051
Without	32 (72.73%)	0.069	With	12 (27.27%)	
With	12 (27.27%)		T stage		
Other organ metastasis			T1	5 (11.36%)	
Without	24 (54.55%)	0.805	T2	14 (31.82%)	
With	20 (45.45%)		T3	9 (20.45%)	0.823
Number of brain metastatic lesions			T4	16 (36.37%)	
1	23 (52.27%)	0.511	N stage		
≥2	21 (47.73%)		N0	6 (13.64%)	
Maximum diameter of BM (cm)			N1	1 (2.27%)	
≤2	31 (70.45%)	0.700	N2	18 (40.90%)	0.033^{*}
>2	13 (29.55%)		N3	19 (43.19%)	
Meningeal metastasis			EGFR		
Without	35(79.55%)	0.269	Wild type	8 (18.18%)	0.007^{**}
With	9 (20.45%)		Mutant	21 (47.73%)	

$^{*}P<0.05$, $^{**}P<0.01$
